# *Plasmodium *Cysteine Repeat Modular Proteins 3 and 4 are essential for malaria parasite transmission from the mosquito to the host

**DOI:** 10.1186/1475-2875-10-71

**Published:** 2011-03-31

**Authors:** Bruno Douradinha, Kevin D Augustijn, Sally G Moore, Jai Ramesar, Maria M Mota, Andrew P Waters, Chris J Janse, Joanne Thompson

**Affiliations:** 1 Malaria Unit, Instituto de Medicina Molecular, Faculdade de Medicina de Lisboa, Av. Prof. Egas Moniz, 1649-028 Lisbon, Portugal; 2 Department of Parasitology, Leiden University Medical Centre, Postbus 9600 RC, Netherlands; 3 Institute of Immunology and Infection Research, School of Biological Sciences, University of Edinburgh, Edinburgh EH9 3JT, UK; 4 Glasgow Biomedical Research Centre, University of Glasgow, Glasgow G12 8QQ, UK; 5 VU University Amsterdam, De Boelelaan 1081 1081 HV Amsterdam, Netherlands; 6 Molecular Vaccinology Lab, Queensland Institute for Medical Research, 300 Herston Rd, Herston, Australia

## Abstract

**Background:**

The *Plasmodium *Cysteine Repeat Modular Proteins (PCRMP) are a family of four conserved proteins of malaria parasites, that contain a number of motifs implicated in host-parasite interactions. Analysis of mutants of the rodent parasite *Plasmodium berghei *lacking expression of PCRMP1 or 2 showed that these proteins are essential for targeting of *P. berghei *sporozoites to the mosquito salivary gland and, hence, for transmission from the mosquito to the mouse.

**Methods:**

In this work, the role of the remaining PCRMP family members, PCRMP3 and 4, has been investigated throughout the *Plasmodium *life cycle by generation and analysis of *P. berghei *gene deletion mutants, Δ*pcrmp*3 and Δ*pcrmp*4. The role of PCRMP members during the transmission and hepatic stages of the *Plasmodium *lifecycle has been evaluated by light- and electron microscopy and by analysis of liver stage development in HEPG2 cells *in vitro *and by infecting mice with mutant sporozoites. In addition, mice were immunized with live Δ*pcrmp*3 and Δ*pcrmp*4 sporozoites to evaluate their immunization potential as a genetically-attenuated parasite-based vaccine.

**Results:**

Disruption of *pcrmp3 *and *pcrmp4 *in *P. berghei *revealed that they are also essential for transmission of the parasite through the mosquito vector, although acting in a distinct way to *pbcrmp1 *and *2*. Mutants lacking expression of PCRMP3 or PCRMP4 show normal blood stage development and oocyst formation in the mosquito and develop into morphologically normal sporozoites, but these have a defect in egress from oocysts and do not enter the salivary glands. Sporozoites extracted from oocysts perform gliding motility and invade and infect hepatocytes but do not undergo further development and proliferation. Furthermore, the study shows that immunization with Δ*crmp3 *and Δ*crmp4 *sporozoites does not confer protective immunity upon subsequent challenge.

**Conclusions:**

PCRMP3 and 4 play multiple roles during the *Plasmodium *life cycle; they are essential for the establishment of sporozoite infection in the mosquito salivary gland, and subsequently for development in hepatocytes. However, although Δ*pcrmp3 *and Δ*pcrmp4 *parasites are completely growth-impaired in the liver, immunization with live sporozoites does not induce the protective immune responses that have been shown for other genetically-attenuated parasites.

## Background

The malaria parasite, *Plasmodium*, is an obligate intracellular parasite that must recognize, invade and develop within a range of diverse cell-types during its' complex lifecycle whilst evading host clearance mechanism. A family of four conserved proteins, the *Plasmodium *Cysteine Repeat Modular Proteins (PCRMP1-4) that may be involved in several of these processes has recently been described [1]. PCRMPs contain motifs implicated in intercellular interactions or host-protein binding, including Epidermal Growth Factor (EGF-like) and Kringle domains, and a multipass transmembrane domain that is characteristic of integral membrane receptors or channels. Analyses also revealed structural similarities with the ligand binding domains of Tumor Necrosis Factor Receptor (TNFR) superfamily members, suggesting that they may bind host immune molecules and thereby modify the course of the immune response.

It has previously been shown that PCRMP1 and 2 co-localize with the *Plasmodium falciparum *Erythrocyte Membrane Protein 1 (PfEMP1) within structures (the Maurer's Clefts) that traffic exported parasite proteins to the surface of mature blood-stage parasites, and that they are also expressed on the surface of sporozoites. *Pcrmp1 *and *2 *gene knockout parasites of the rodent malaria model, *Plasmodium berghei*, establish normal blood-stage infection and transmit to the mosquito to form oocysts that release large numbers of sporozoites into the mosquito haemocoel. These sporozoites are infectious to the mouse when injected intravenously but are unable to target and invade mosquito salivary glands so do not transmit via infected mosquito bite.

In this work, a similar transgenesis approach was used to analyse the function of the remaining two PCRMP family members, PCRMP3 and 4. The results showed that they also play a role in sporozoite development in the mosquito, although distinct from that of PCRMP1 and 2, and an additional role in intrahepatocytic development. Very little is known of the molecular basis of sporozoite development within the liver after they have invaded the hepatocytes [2] but the proteins involved are under increasing scrutiny since the discovery that *Plasmodium *sporozoites with specific genetic modifications (genetically-attenuated parasites; GAS or GAPs) may be as efficient as radiation-attenuated sporozoites (RAS) in conferring protection against Malaria [3-15]. Since sporozoites that do not express PCRMP3 or 4 are fully growth-arrested in hepatocytes, this study focused on the role of PCRMP3 and 4 in liver-stage parasite development and evaluated the immunization potential of *Δpcrmp3 *and *Δpcrmp4 *sporozoites.

## Methods

### *Plasmodium berghei *parasites and mice

The reference line of *P. berghei *ANKA strain used was cl15cy1 [16]. Animal experiments in the Netherlands were performed after a positive recommendation of the Animal Experiments Committee of the LUMC (ADEC) was issued to the licensee. The Animal Experiment Committees are governed by section 18 of the Experiments on Animals Act and are registered by the Dutch Inspectorate for Health, Protection and Veterinary Public Health, which is part of the Ministry of Health, Welfare and Sport. The Dutch Experiments on Animal Act is established under European guidelines (EU directive no. 86/609/EEC regarding the Protection of Animals used for Experimental and Other Scientific Purposes).

### Generation of Δ*pcrmp3 *and Δ*pcrmp4 *mutant parasites

Two different replacement constructs, pL1082 and pL1083 were made to disrupt *pcrmp3 *and *pcrmp4*, respectively. Target regions of *pcrmp3 *(PBANKA_060670) and *pcrmp4 *(PBANKA_130080) were amplified from genomic DNA of mixed blood stages of *P. berghei *cl15cy1 using the following primers (see Additional file 1, Table S1 for the sequence of the primers): primers L1448 and L1449 for the 5'-target region of *pcrmp3*; L1450 and L1451 for the 3'-target region of *pcrmp3*); L1457 and L1458 for the 5'-target region of *pcrmp4*; L1459 and L1461 for the 3'-target region of *pcrmp4*. Fragments were digested (KpnI, HindIII and EcoRI, BamHI respectively) and ligated into vector pB3D [17] that contains the *Toxoplasma gondii dihydrofolate reductase-thymidylate synthase *(*tgdhfr/ts*) selection cassette. For transfection, pL1082 and pL1083 were linearized with KpnI/BamHI and transfected into purified schizonts of *P. berghei*. Transfection, selection and cloning of mutant parasite lines was performed as described [16] and were carried out in duplicate for each gene, generating *Δpcrmp3a *and *b *and *Δpcrmp4a *and *b*. Correct integration of the construct into the genome of mutant parasites was analysed by Southern blot analysis of digested genomic DNA and/or of FIGE separated chromosomes [16]. Separated chromosomes were hybridized with a DNA-probe specific for the 3'UTR region of *pbdhfr/ts *gene which recognizes the integrated construct in the target locus on chromosome 6 (*pcrmp3*) or on chromosome 13 (*pcrmp4*) and the endogeneous *pbdhfr/ts *gene on chromosome 7. In addition, a probe specific for the selectable marker, the *tgdhfr/ts *gene of the replacement constructs, was used. For Southern analysis of digested DNA, DNA-probes were used recognizing the 5'-regions of the *pcrmp *genes, amplified using the primers L1448, L1449, L1457 and L1458 (Additional file 1, Table S1).

### Determination of growth and multiplication of asexual blood stages

The multiplication rate of asexual blood stages *in vivo*, determined during the cloning procedure, is calculated as follows: the percentage of infected erythrocytes in Swiss OF1 mice (OF1-ico, Construct 242; age 6 weeks old; Charles River) injected with a single parasite is determined at day 8 to 11 by counting Giemsa stained blood films. The mean asexual multiplication rate per 24 hour is then calculated assuming a total of 1.2 × 10^10 ^erythrocytes per mouse [18,19]. The percentage of infected erythrocytes in mice infected with reference lines of the *P. berghei *ANKA strain consistently ranged between 0.5 and 2% at day 8 after infection, resulting in a mean multiplication rate of 10 per 24 h [18,19]. Such an analysis is a sensitive method to quantify differences in growth rate during the early phase of *in vivo *infections when the availability of suitable host cells (reticulocytes) is not a limiting factor [19].

### Transmission electron microscopy

Guts from mosquitoes infected with WT, *Δpcrmp3 *and *Δpcrmp4 *parasites were dissected as previously described [20], and treated for Electron Microscopy observation as reported in [21,22]. Samples were observed in a TEM Jeol JEM - 100 CX II microscope.

### Gliding and cellular assays for Δ*pcrmp3 *and Δ*pcrmp4 *sporozoites

WT and mutant sporozoites were obtained by dissection of infected mosquitoes 17-25 days after the infectious blood meal from salivary glands or guts respectively, as previously described [20,23]. To assess sporozoite motility, circumsporozoite protein (CSP) gliding trails were visualized by staining with a monoclonal antibody against CSP [24,25]. The hepatoma cell line HepG2 (ATCC, HB8065), which is efficiently infected by *P. berghei *parasites and sustains their complete development, was used for *in vitro *hepatocyte invasion assays [26]. Cells were maintained in DMEM supplemented with 10% Fetal Calf Serum and 1% Penicillin-Streptomycin (complete DMEM), in an atmosphere containing 5% CO_2 _and were periodically tested for mycoplasma infections, as previously described [27]. For migration and infection assays, *P. berghei *sporozoites were added to cell monolayers seeded 24 hours earlier on coverslips in complete DMEM and used when confluence was ~80-90%. Sporozoite migration through cells was quantified by detection of parasite-wounded hepatocytes using a cell-impermeant fluorescent tracer macromolecule, rhodamin-dextran [24] and staining for CSP. Migration through host cells was quantified as the percentage of dextran-positive non-infected cells, and the number of sporozoites that reach a final hepatocyte for infection and further development, as the percentage of parasites inside dextran-negative cells [24]. Intrahepatocytic development was measured at 24, 48 and 56 hours after infection, stained against parasite HSP70 [28] and with the DNA dye diamidino-phenyl-indole (DAPI). Migration, infection and intrahepatocytic development was imaged using Metamorph software (Molecular Devices, Sunnyvale, California, USA). All assays were performed at least twice and results were analysed by paired Ttest, two tailed.

### Immunizations

6-8 week old female BALB/c (H-2K^d^) and C57BL6 (H-2K^b^) were supplied by Haarlan, UK. Mice were immunized intravenously with either *Δpcrmp3 *or *Δpcrmp4 *sporozoites, according to regimens proven effective for RAS and other GAPs; a single dose of 50,000 of mutant sporozoites for BALB/c and three doses of 50,000/20,000/20,000 of mutant sporozoites for C57BL6, weekly apart [7,8,29]. Immunized and naïve control group mice were challenged 10 days later with 10,000 WT *P. berghei *sporozoites. Parasitaemia was measured by blood smears and Giemsa staining from day 4 onwards [7,8].

## Results

### PCRMP3 and 4 are expressed in blood and mosquito-stage parasites

The *P. berghei *gene models of PCRMP3 and 4 are PBANKA_060670 and PBANKA_130080 respectively. PCRMP3 and 4 from *P. berghei, Plasmodium vivax, Plasmodium knowlesi *and *P. falciparum *are shown in alignment in Additional File 2. The open reading frame (ORF) of the 5' region of *pbcrmp3 *and *4*, containing 2 and 1 introns respectively, was verified by RT-PCR of cDNA prepared from blood infected with mixed asexual-stage parasites and gametocytes. Peptides and mRNA (expressed sequenced tags) from PfCRMP3 (PlasmoDB identifier; PFL0410w) and PfCRMP4 (PF14_0722) have been detected in *P. falciparum *blood-stage parasites [30,31], and microarray data from *Plasmodium yoelli, P. falciparum *and *P. vivax *(available at PlasmoDB) are also consistent with a blood-stage pattern of expression. In addition, peptides and mRNA of PCRMP3 and 4 have been detected in *P. berghei, P. yoelli *and *P. falciparum *mosquito midgut- and salivary gland-sporozoites indicating that, like PCRMP1 and 2, these family members may also play a role during transmission.

### Generation of *P. berghei *parasite lines with disrupted *pcrmp3 *and *pcrmp4 *genes

**Figure 1:**
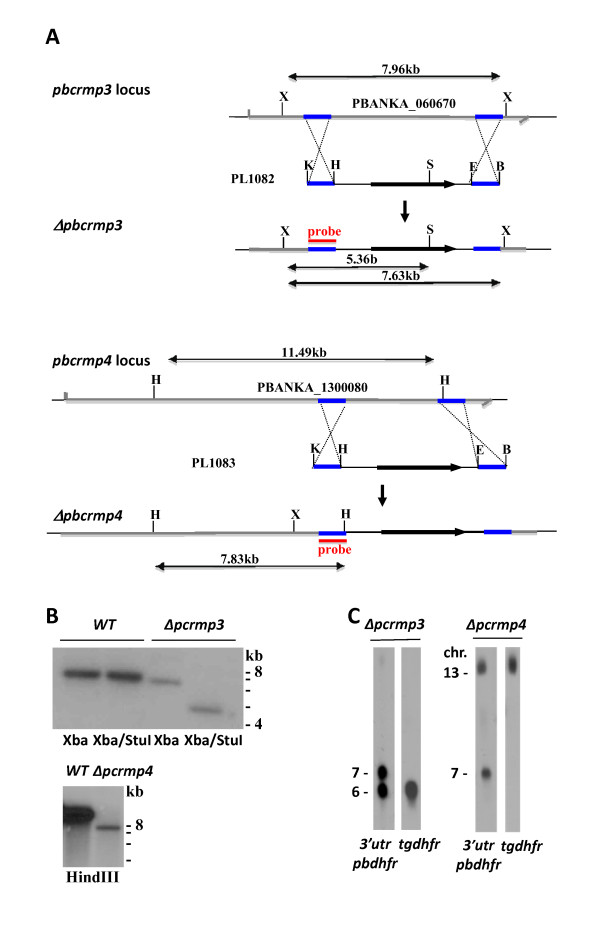
**Generation of *Δpcrmp3 *and *Δpcrmp4 *parasites**. ***A. ***Transfection of *P. berghei *parasites with constructs PL1082 or PL1083 containing the *tgdhfr *gene flanked by target sequences for homologous recombination within the *pbcrmb3 *or *4 *loci generated *Δpcrmp3*a and b and *Δpcrmp4*a and b respectively. Fragment sizes produced by restriction enzyme digestion are shown in kb. X, Xho1; K, Kpn1; H, HindIII; S, StuI; E, EcoR1, B, BamH1. ***B. ***Southern blots of genomic DNA from WT and *Δpcrmp3 *or *4 *clones digested with enzymes indicated were hybridized with specific probes within the 5' target region of *pcrmp3 *or *4*. ***C. ***Chromosome analysis of *Δpcrmp3 *and *4 *parasite clones. Chromosomes were hybridized to probes specific for the *P. berghei dhfr/ts *3'UTR region or for *tgdhfr *within the selection cassette, as indicated. The *pbdhfr/ts *probe hybridizes to the *P. berghei dhfr/ts *gene on chromosome 7. Both probes hybridize to the site of integration of constructs PL1082 in the *pbcrmp3 *locus on chromosome 6 in *Δpcrmp3 *or the site of integration of PL1083 in the *pbcrmp4 *locus on chromosome 13 in *Δpcrmp4*.

To examine the role of PCRMP3 and PCRMP4 during the *Plasmodium *life cycle, Δ*pcrmp3 *and Δ*pcrmp4 *mutant parasite lines were generated. The genes were disrupted using standard genetic modification technologies aimed at disruption of the gene via double cross-over integration [16]. In these experiments, integration of the construct deletes a fragment of 4965 bp or 3625 bp of the ORF of *pbcrmp3 *or *pbcrmp4 *respectively encoding the Cysteine Repeat Modular and mTM domains. After transfection, selected pyrimethamine-resistant parasites were cloned for further genotype and phenotype analysis. Correct integration of the constructs in the cloned Δ*pcrmp3 *and Δ*pcrmp4 *parasites was shown by Southern analysis of FIGE separated chromosomes and of digested genomic DNA (Figure 1). Transfection, selection and cloning of mutant parasite lines was carried out in duplicate for each gene. In each case, growth and development of blood- and mosquito stage parasites appeared the same in both mutant parasite lines, and one of each was chosen for further analysis.

### Δ*pcrmp3 *and Δ*pcrmp4 *parasites show normal blood stage growth but defects in sporozoite release and salivary gland invasion

The generation of mutants with a disrupted *pcrmp3 *or *pcrmp4 *gene demonstrates that they are not essential for survival of blood stage parasites. To determine if *pcrmp3 *or *pcrmp4 *gene disruption has an effect on parasite growth rate during blood stages, the *in vivo *growth was analysed during the experiments in which the mutant parasites were cloned by limiting dilution. Such an analysis is a sensitive method to quantify differences in growth during the early phase of *in vivo *infections. In these experiments, no effect on the growth rate of the mutant parasites was observed. All mice infected with a single *Δpcrmp3 *or *Δpcrmp4 *parasite showed a parasitaemia level of 0.5-2% at 8 days post infection; therefore these mutant parasites have an asexual multiplication rate, similar to wildtype (WT) parasites, of 10 per 24 hours. Mutant parasites also produced gametocytes that were infectious to *Anopheles stephensi *mosquitoes, and produced oocysts in comparable numbers to WT parasites following transmission (WT, 176 ± 74 oocysts/infected mosquito; *Δpcrmp3*, 229 ± 83; *Δpcrmp4*, 192 ± 106).

*Plasmodium berghei *sporogony occurs in an encapsulated oocyst attached to the mosquito midgut epithelium. A mature oocyst is a spherical cell, 30-40 μm in diameter, limited by a plasma membrane and a thick capsule. It contains numerous dividing nuclei and cytoplasmic membranes that segregate into individual sporozoites from days 10-12 after an infective blood meal. In mosquitoes housed at 18-19°C, sporozoites begin to exit oocysts from day 11-12 and rapidly target and invade the salivary glands. At day 21 of mosquito infection, salivary glands of mosquitoes infected with *Δpcrmp3 *or *Δpcrmp4 *parasites contained no sporozoites whereas WT parasite-infected mosquito salivary glands contained an average of 1742 sporozoites (n = 36). Many *Δpcrmp3 *or *Δpcrmp4 *sporozoites were visible within oocysts at day 21 indicating a reduced ability to egress (Figure 2.1). To determine whether mutant sporozoites are unable to egress due to defective development, the morphology of sporozoite-containing oocysts was analysed at two timepoints after infective blood meal by Electron Microscopy. At day 10, no difference was observed between WT, *Δpcrmp3 *or *Δpcrmp4 *oocysts; all contained numerous nuclei within forming sporozoites (Figure 2.2) At day 18, WT oocysts contained few nuclei and appeared degenerate or undergoing melanization. In contrast, at day 18 the membranes of mutant oocyst appeared intact and full of morphologically-normal, segregated sporozoites.

**Figure 2:**
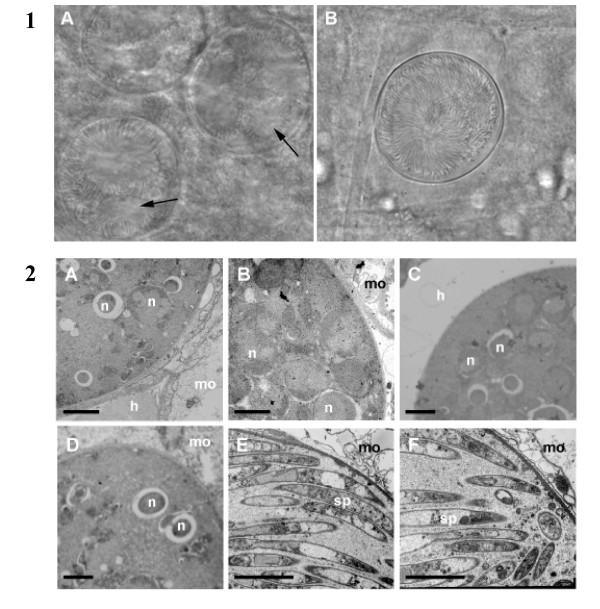
***Δpcrmp3 *and *Δpcrmp4 *sporozoites develop normally but remain within mature oocysts**. 1) At day 18 post infection, WT oocysts (A) contain few sporozoites and show areas of degradation (arrowed), whilst sporozoites within *Δpcrmp3 *oocysts (B) are radially-aligned and are retained within an intact cell wall. Scale = 100 mm. 2) WT (A), *Δpcrmp3 *(B) and *Δpcrmp4 *(C) oocysts are found in mosquito guts 10 days post infection, developing between the mosquito epithelium (mo) and haemolymph (h). Several nuclei (n) in developing sporozoites are visible. No morphological differences are observed. At day 18 post infection, WT oocysts contain few nuclei (D) whilst most *Δpcrmp3 *(E) and *Δpcrmp4 *(F) mature oocysts are full of sporozoites (sp). Scale = 5 μm.

### *Δpcrmp3 *and *Δpcrmp4 *sporozoites show normal gliding motility and hepatocyte traversal *in vitro*

Sporozoites undergo gliding motility, characterized by circular movements across the substrate that propel the parasite forward and leaves a trail of CSP [24,32,33]. Oocyst-derived *Δpcrmp3 *and *Δpcrmp4 *sporozoites deposit several circular trails (3.7 ± 1.6 and 3.7 ± 1.5 respectively; n = 6) per sporozoite) and show comparable levels of gliding motility to WT sporozoites (3.5 ± 1.5; n = 8) of the same age extracted from infected salivary glands. Following transmission, sporozoites migrate through several hepatocytes before invading and establishing infection in a final hepatocyte; a feature dependent on sporozoite gliding motility [24,32]. Sporozoites migrate through hepatocytes by disrupting their membrane, which is rapidly repaired, but enter the hepatocyte in which they will develop with the formation of a parasitophorous vacuole (PV). Cells that have been traversed can be visualized using a rhodamine-dextran tracer that enters the cell immediately after membrane disruption and becomes trapped inside after its' repair [24,32]. To determine whether mutant parasites migrate through hepatocytes, rhodamine dextran-positive, uninfected hepatocytes that had been traversed by WT salivary gland sporozoites and *Δpcrmp3 *and *Δpcrmp4 *sporozoites extracted from oocysts at day 21 post infection were quantified. The results show that traversal of either of the mutant parasites through HepG2 cells does not differ significantly (*P *< 0.05) from WT sporozoites (Figure 3.1).

**Figure 3:**
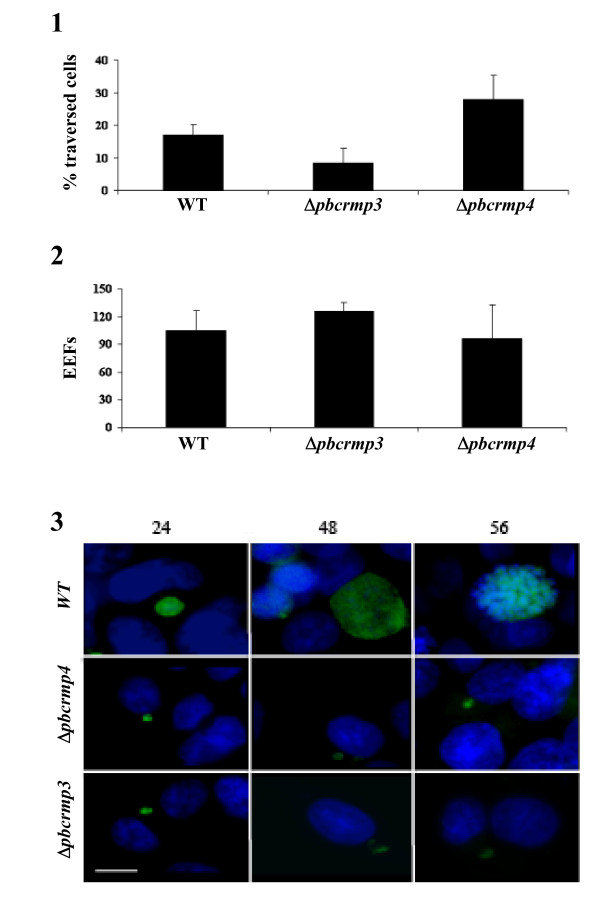
***Δpcrmp3 *and *Δpcrmp4 *sporozoites migrate through and infect HepG2 cells**. 1) HepG2 cells were incubated with WT salivary gland sporozoites or with *Δpcrmp3 *or *Δpcrmp4 *oocyst-derived sporozoites in the presence of rhodamine dextran. Migration rates are expressed as the percentage of uninfected traversed (rhodamine dextran +ve) cells ± the standard deviation. 2) HepG2 cells were incubated with WT salivary gland sporozoites or with *Δpcrmp3 *or *Δpcrmp4 *oocyst-derived sporozoites and the development of EEFs was quantified at 24 hours. Infected cells are expressed as the mean ± the standard deviation for each coverslip. 3) WT parasites show normal development in EEFs at 24 hours post-infection and develop into trophozites at 48 hours and schizonts at 56 hours. *Δpcrmp3 *and *Δpcrmp4 *parasites establish infection at 24 hours but remain small with irregular shapes. Fluorescence pictures of EEF (green) and nuclei (blue). Scale = 5 μm.

### *Δpcrmp3 *and *Δpcrmp4 *sporozoites have impaired intrahepatocytic growth

Following infection of the hepatocyte, sporozoites develop into exo-erythrocytic forms (EEFs), forming trophozoites, schizonts and merozoites, which are released into the bloodstream. To determine whether *Δpcrmp3 *and *Δpcrmp4 *sporozoites develop within hepatocytes, WT salivary gland sporozoites and mutant sporozoites extracted from oocysts at day 21 post-infection were incubated with HepG2 cells, and the number of EEFs was quantified (Figure 3.2). No significant differences were observed in the level of infection. WT and mutant parasites formed similar numbers of EEFs but *Δpcrmp3 *and *Δpcrmp4 *EEFs were smaller and aberrant at 24 hours and did not undergo any further growth or nuclear division (Figure 3.3). Mutant EEFs persisted up to 56 hrs post invasion but infected hepatocytes showed no nuclear condensation or fragmentation or other morphological indications of apoptosis.

*Δpcrmp3 *and *Δpcrmp4 *sporozoites do not confer protective immunity to mice.

**Figure 4:**
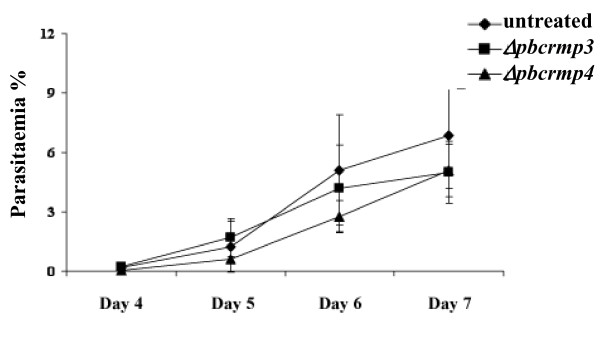
**C57BL/6 mice immunized with *Δpcrmp3 *(black square) or *Δpcrmp4 *(black triangle) sporozoites develop a patent infection with the same kinetics as unimmunized mice (black diamond) upon subsequent infection with WT sporozoites**.

Immunization with sporozoites that are attenuated by irradiation or genetic modification and that infect hepatocytes but arrest development at an early stage can confer protection against subsequent challenge with infectious sporozoites [3,5-15,34]. Since *Δpcrmp3 *and *Δpcrmp4 *parasites also arrest during development in hepatocyes, it was, therefore, determined whether they can confer protection in a similar way. Balb/c and C57BL/6 mice were immunized intravenously with *Δpcrmp3 *or *Δpcrmp4 *sporozoites extracted from mature oocysts at day 21 post-infection. No immunized mice developed patent bloodstage infections. Upon challenge with WT sporozoites, however, all immunized mice developed a blood-stage parasitemia with similar pre-patent periods and parasitaemia levels as naïve mice control (Figure 4) and showed no protective immunity (Table 1).

**Table 1:** Immunization with *Δpcrmp3 *or *Δpcrmp4 *sporozoites does not protect Balb/c or C57BL/6 mice against subsequent challenge with WT sporozoites

	*Δpcrmp3 *or *Δpcrmp4 *sporozoites			WT	#protected/# challenged		
**Mouse Strain**	**Immunization**	**Boost I**	**Boost II**	**Sporozoite Challenge**	**Control**	***Δpbcrmp3***	***Δpbcrmp4***

Balb/c	50 000	-	-	10 000	0/6 (0)	0/6 (0)	0/6 (0)

C57Bl/6	50 000	20 000	20 000	10 000	0/6 (0)	0/6 (0)	0/6 (0)

Groups of mice were immunized *i.v. *with 50,000 *Δpcrmp3 *or *Δpcrmp4 *sporozoites isolated from several mosquitoes. Boosts were performed with 20,000 sporozoites at weekly intervals. Mice were challenged with 10,000 WT sporozoites 10 days after immunization (Balb/c) or second boost (C57BL/6), and infections were monitored by counting of giemsa-stained blood smears. All mice became positive on day 4 after challenge.

## Discussion

This study demonstrated that the PCRMP family members, PbCRMP3 and 4, play roles during parasite development in the mosquito and in the liver of the vertebrate host that are distinct from family members PCRMP1 and 2. In the mosquito, both PCRMP3 and 4 are involved in the process of egress of morphologically mature sporozoites from oocysts. Mutant sporozoites lacking expression of these proteins are released inefficiently from oocysts and do not invade the mosquito salivary gland, so are unable to transmit to the mammalian host. This phenotype shows some similarity with that of parasites containing mutations in the conserved region II plus of Circumsporozoite Protein (CSP; PBANKA_040320) and in parasite mutants lacking expression of Egress Cysteine Protease 1 (ECP1; PBANKA_030470), PSOP9 (PBANKA_070190) and Sporozoite Invasion-associated Protein 1, (SIAP-1; PBANKA_100620) [35-38]. However, unlike CSP, PSOP9 and SIAP-1 mutants, *Δpcrmp3 *and *Δpcrmp4 *sporozoites developing within oocysts not only appear normal at the ultrastructural level but are also able to undergo gliding motility and hepatocyte invasion, suggesting that the defect does not lie indirectly in incorrect assembly or maturation of components of the motility machinery (such as CSP). In this respect, the mutant phenotype observed most closely resembles that of *Δpcrmp1 *and *Δpcrmp2 *parasites, although occurring at an earlier stage of sporozoite development. Although further experiments are required to determine the precise role of PCRMP3 and 4 in oocyst egress, it is intriguing that the small numbers of sporozoites that are released from oocysts do not invade the salivary glands. As with PCRMP1 and 2, this may indicate a defect in targeting or initiation of motility in the physiological environment of the midgut, and thus a possible role in the signal transduction pathways that are known to operate in all parasite stages undergoing migration.

Sporozoites of *Δpcrmp3 *and *Δpcrmp4 *extracted from oocysts perform gliding motility *in vitro *and are able to traverse through several hepatocytes before committing to infection in a final hepatocyte with levels of infectivity that are similar to those of WT sporozoites extracted from salivary glands. It has previously been thought that oocyst and haemolymph sporozoites are intrinsically less infectious than those that reach the salivary gland, and that infectivity is enhanced by mechanisms that are activated during salivary gland invasion [39,40]. Since it is difficult to obtain sufficient numbers of mature WT sporozoites from the haemolymph, however, these conclusions have largely arisen from experiments using sporozoites extracted from oocysts at day 14 of development. The results from this and a previous study [1] have shown that mature sporozoites lacking expression of PCRMPs extracted from oocysts and the haemolymph at day 18-21 of development show levels of gliding motility and hepatocyte infectivity that are comparable to WT salivary gland sporozoites of the same age. These observations, therefore, support an alternative view [41]; that the principal factors affecting sporozoite infectivity *in vitro *are age and maturity, rather than a further maturation process within the salivary gland. It should be noted, however, that parasite transmission to a host via mosquito bite is a more complex process requiring sporozoite migration within the skin of the host at the injection site [33,42,43] before entry into a blood vessel and transport to the liver. Thus, further experiments are needed to establish whether sporozoite maturation within the Salivary Gland is important for infection *in vivo*, and whether PCRMP3 and 4 play a role in this process.

These studies show that PCRMP3 and 4 play an additional role during parasite development within hepatocytes. *Δpcrmp3 *and *Δpcrmp4 *sporozoites traverse and infect hepatocytes at WT rates but arrest development at a very early stage and are unable to develop into the replicating liver-stage forms. This phenotype differs from that of parasites lacking expression of PCRMP1 and 2 that are not only able to initiate infection within the liver but undergo further development and establish blood-stage infection [1]. Immunization with genetically-attenuated parasites that show a similar growth arrest in the liver-stages may confer protection against subsequent infection with WT sporozoites, (reviewed in [8,44]). However, immunization with *Δpcrmp3 *and *Δpcrmp4 *sporozoites did not result in protective immunity and challenge of immunized mice with wild type parasites showed normal liver stage development as determined by the length of the prepatent period. Interestingly, it was observed that growth-arrested *Δpcrmp3 *and *Δpcrmp4 *parasites persist in infected hepatocytes for prolonged periods but no evidence was found that infected hepatocytes undergo apoptosis. Antigen presentation is likely to be less efficient in the absence of infected-hepatocyte apoptosis which provides a huge array of antigens to antigen-presenting cells [45] and it has been suggested that this mechanism plays an important role in attenuated sporozoite-mediated protection [5,7,8,46]. It is possible, therefore, that the lack of protection conferred by *Δpcrmp3 *and *Δpcrmp4 *sporozoites is associated with the lack of induction of apoptosis.

## Conclusions

In this work, the role of PCRMP3 and 4 was addressed during different stages of the *Plasmodium *life cycle. The results show that disruption of the respective genes led to blocking of transmission of *P. berghei *parasites from the mosquito, where they remain trapped inside oocysts, to the mouse. The infectivity of *Δpcrmp3 *and *Δpcrmp4 *sporozoites to hepatocytes mirrors that or wild type sporozoites derived from salivary glands. This observation implies that popular concept of "enhanced" infectivity of salivary gland sporozoites is more grounded in age and maturity of the sporozoite rather than its activation due to successful colonization of the salivary gland. *Δpcrmp3 *and *Δpcrmp4 *sporozoites are also impaired in maturing efficiently in the liver implying a further role for these proteins during intrahepatocytic development.

## Competing interests

The authors declare that they have no competing interests.

## Authors' contributions

BD performed the liver stage and EM experiments, assessment of immunization potential, and participated in study design and drafting of the manuscript. KDA and JR participated in generation and characterization of *Δpcrmp3 *and *Δpcrmp4 *parasites. SGM assisted in all aspects of the experimental work. APW and MMM participated in study design. CJJ participated in study design, generation and characterization of mutant parasites and writing of the paper. JT conceived and designed the study, participated in the characterization of mutant parasites and wrote the manuscript.

All authors have read and approved the final manuscript.

## Supplementary Material

Additional file 1 **Table S1: Primers used in this study**. Click here for file

Additional file 2 A) Alignment of PCRMP3 from *P. vivax *(PvCRMP3)*, P. knowlesi *(PkCRMP3)*, P. falciparum *(PfCRMP3) *and P. berghei *(PbCRMP3); B) Alignment of PCRMP4 from *P. vivax *(PvCRMP4)*, P. knowlesi *(PkCRMP4)*, P. falciparum *(PfCRMP4) *and P. berghei *(PbCRMP4). Click here for file
