# Supplementary material for: Plasmodium Cysteine Repeat Modular Proteins 3 and 4 are essential for malaria parasite transmission from the mosquito to the host
Source: Malar J. 2011 Mar 31;10:71. doi: 10.1186/1475-2875-10-71 (PMC3083381; doi:10.1186/1475-2875-10-71)
Supplement: Additional file 1 — Table S1: Primers used in this study. [file 1475-2875-10-71-S1.DOC]

Table S1: Primers used in this study

| **sequence** | **Name** | **Additional information** |
| --- | --- | --- |
| CACCGAGGTACCGTGATAGCGGCTTAGACCAGCC | L1448 (KpnI); 5'target forward | Pcrmp3: targeting construct (5’) and probe for Southern analysis |
| AGTTTAAAGCTTCATAACTTTTGGCTCCAGTTACAGTAC | L1449 (HindIII); 5'target reverse | Pcrmp3: targeting construct (5’) and probe for Southern analysis |
| GTGTTTGAATTCCATTTTCCGTTCACACTTATGAGTATATTAG | L1450 (EcoRI); 3'target forward | Pcrmp3: targeting construct (3’) |
| TTTCGGATCCTTCTCATTAAAAAATATGGAACATGAAGG | L1451 (BamHI); 3'target reverse | Pcrmp3: targeting construct (3’) |
| GTAAATGGTACCTTGGCATCTTCTATTTTCGATTGTG | L1457 (KpnI); 5'target forward | Pcrmp4: targeting construct (5’) and probe for Southern analysis |
| ATCTTCAAGCTTCTTTATTGGTACACCATTCTGAACG | L1458 (HindIII); 5'target reverse | Pcrmp4: targeting construct (5’) and probe for Southern analysis |
| TCAAAGAATTCATATGGTACAAACTATGGGATACGATGACCAC | L1459 (EcoRI/NdeI); 3'target forward | Pcrmp4: targeting construct (3’) |
| TTACCGGATCCATTTGAGTCTTCCCTACTGTTTTGCTC | L1461 (BamHI); 3'target reverse | Pcrmp4: targeting construct (3’) |
