# Supplementary material for: Plasmodium Cysteine Repeat Modular Proteins 3 and 4 are essential for malaria parasite transmission from the mosquito to the host
Source: Malar J. 2011 Mar 31;10:71. doi: 10.1186/1475-2875-10-71 (PMC3083381; doi:10.1186/1475-2875-10-71)
Supplement: Additional file 2 — A) Alignment of PCRMP3 from P. vivax (PvCRMP3), P. knowlesi (PkCRMP3), P. falciparum (PfCRMP3) and P. berghei (PbCRMP3); B) Alignment of PCRMP4 from P. vivax (PvCRMP4), P. knowlesi (PkCRMP4), P. falciparum (PfCRMP4) and P. berghei (PbCRMP4). [file 1475-2875-10-71-S2.DOC]

**Supplementary Figure 1;**

**A) Alignment of PCRMP3 from *P. vivax* (PvCRMP3)*, P. knowlesi* (PkCRMP3)*, P. falciparum* (PfCRMP3) *and P. berghei* (PbCRMP3)*.***

PvCRMP3 -MLIFFFFCFLLFFALPPDTTTERGQQMYEMYGSPSYSLDSDLCVADGSTRCCPSCALPF 59

PkCRMP3 -MLIFFCCCFILFSSLSHDPTG-RVQQL---YGNLCYNSNSGLFVAHNPARCCTSCAGSF 55

PfCRMP3 MKKIYLSICLMIIVIFKIFKKK---QHI---MDKHFYDRN--FYKKHNHGFTCNLFLILY 52

PbCRMP3 -MEIRAVILFISFFAYNANIIIKN-EFV---KAKQYYSYEYNINRSSNYLLPHTHGIYAF 55

* :: : : : . *. : : . :

PvCRMP3 SPG-REVLSASIFCHHDEFIKR-----------G---NCRFV-------GHLTDEQWCSQ 97

PkCRMP3 SPGSREILSFSNFYHHDEFIKRRIYPFQIWISHGHIHKCAYIYMYSYVCAIFSPFARCNE 115

PfCRMP3 IPYKRSLCYDTLFN---QMIKE---------------------------STFP-----SK 77

PbCRMP3 SLDKTTPIFSTFVLNNNKFFIRNYYKFEKYSNSCIYFNVRYFQLKIFHSNNIEIYSRHSK 115

: . ::: . : .:

PvCRMP3 PNLTPHRAK--------------------------------------------------E 107

PkCRMP3 PNLTQREAQTGKYTRINVPRGGGNPLQNKLKEKPLSLPSLLRLGGWKIVQPSAAYTSEEE 175

PfCRMP3 INVTYNLRY--------------------------------------------------H 87

PbCRMP3 QNKTIFIDIILSNVLNKKRAHSNN----------------------------YLYHLTID 147

* * .

PvCRMP3 GAPLVAAGEEKGE------------MPPRESLISRP---------LPSR--PLPSRPLTI 144

PkCRMP3 GSPLVHLRRNKAKRVNKKVKIKIITITVRTATMATTTVALVVMEMLPGEE-SLIKRIFTI 234

PfCRMP3 LCPFFSSNNNITKIIN---------NTISRVKIYNN---------FPFS--VTGTTLKSI 127

PbCRMP3 DYKFVKHKQEGCNAENIISTQERIIQVNKKTKYKSNKAYGVVSFFIDLYKSIQNKTIDCV 207

:. .: : : . :

PvCRMP3 SRREPHVQPDSASTSRKQ-TRRCLAEFINV-------------ETSPKGEKKGD-----A 185

PkCRMP3 YRRVPYVHPWSKVTSKKQ-TRRCLAEFINL-------------DTSPKGEKKAS-----G 275

PfCRMP3 IIKYPNKRYLSERKYIEQNQKKSLKKLKHD-------------EIKKVGERKKE-----K 169

PbCRMP3 LSKNENNGSFLKVMDGFKNNKCLFISATHLPSIYYDYFNKNYGNITSKKRKKRDLEEIIN 267

: : : : . : : . .:* .

PvCRMP3 VGGKFQRGSPNDYLAGKQNREEGARNVYR-QEPSEGSGYEV------ESEPSKDVTTSLK 238

PkCRMP3 VEGKYRGETPNDYLVGEKNREKNTKNIHM-QEPSKSSGYHV------ESTPSRDISTSLK 328

PfCRMP3 KKVRYSSYN-KSYLNDEKNTFDNLHSAKY-ISKNRIIKYKINKIKKIEENVFKKINTKKH 227

PbCRMP3 MGNIFHQVDKKINIATKSHESNRTINRKRDVYKSSKNGSNIQ-----NQNNIKKIKYSKK 322

: : : :.: . . . .: :. :.:. . :

PvCRMP3 WLFLNKK-KNKGEENVRNNLSKNIEHTS---EFIEKF-NPMEKP-TLPHILADQMFIITK 292

PkCRMP3 SLFLNRK-KKKGEKNKRNNLSKNIGHTA---EFIEEQ-NPMDKS-PLTHILADQMFIRTK 382

PfCRMP3 HINKNIKLESSGKKESKININNIIIHKNKDRHLMNNT-NFVENPVTNDYIIYKQEFIRSV 286

PbCRMP3 SSTKFLKSRFLDKKMYYSNLSKIKNNILSTIHLNDRLSNNLNNS-RKIQFTPNQTFIRTK 381

* . .:: *:.: : .: :. * :::. : .* ** :

PvCRMP3 NLLSTMEIKGNGLDKFKYQSILIYDSFENCKGTVRAQVYIQDVSPFRILTKNFMINKLGM 352

PkCRMP3 NQMTSIQIKGNKLNNFKYKNILIYDSFENCKGNILEKVSVKEISPFYILTETFMINKLGM 442

PfCRMP3 NTLTSLKLYGMNLNYLEYQILTIYDSTDECSGTKLAEINILEKYDSYMITDSFKIKKVGL 346

PbCRMP3 NVLISLKIYGSINDNVYNGSIYIYKIYGECDGFVIAKVKIIDITSLYIITKHFEINNIGT 441

* : :::: * : . : **. :*.* :: : : ::*. * *:::*

PvCRMP3 FSICIEMEEVKY-AALLKVQSNLIKQITPPNEASTVSAFNCALGKSAMLMPEESKVGIVE 411

PkCRMP3 FSICIEREEPEY-VALLKIQKNLIEQITPLNDVSTVSVFSCNLGESSIYIPEENLVRVVK 501

PfCRMP3 FLICITLTSVEY-VGVLKVQNNLIDDFDSIINNNNISTFNCSTDAKSILIYSKERIINLN 405

PbCRMP3 FSICFNTNDTYIPIGKLKIQNNSIEQFDIISNENYISTFNCPINESSILLHINSHYGAIY 501

* **: . . **:*.* *.:: : . :*.*.* . .:: : :. :

PvCRMP3 KIG-ENLSQKSYP-YNILGKILWKEIKILACSSSEN-YIVILEMNYIIVVNSHLNAVHEI 468

PkCRMP3 KVG-ENLSQKTYS-YDNLGKVLMKEIKILACSSTKN-YAVFLEMNYIIVVNSSMNTVYEV 558

PfCRMP3 TINNEEIKPEEFINLSKYSEYLQGDISLLACCVNSNGYVVVLDKDVIFVLYN--KILKEI 463

PbCRMP3 ADTNGKLSYITNK-HSEIEKKVKSKRYSIACSSSGN-YIAILQKQYVFIVEKNLKFSGEI 559

::. . : : . :**. . * * ..*: : :::: . : *:

PvCRMP3 ITHFLTKPVGIFLDNHIIYVTDADRKNVFRYMSEHIL-KTTAASLVFLDGS--------- 518

PkCRMP3 ISHFLTKPVGIFLDNHIIYVTDADRKDIFRYTSEYIH-QQISSSLTFLDQTGQISHASQM 617

PfCRMP3 IIHFLKNPIAIYLYNHTIFITDAERKNIFRFVSKYIL-KKTSKKLTPIWNY--------- 513

PbCRMP3 IAHFLDNPISIFLDRNIIYITDASRKNIFRYISVHITNKNRSRQLSLLDIR--------- 610

* *** :*:.*:* .: *::***.**::**: * :* : : .* :

PvCRMP3 ---EQGEGGNGVKAPPPEYHTPGETNGRRTYARKKK-RSYLSSLGRIFLEEKRSDHTD-- 572

PkCRMP3 SEMEQGIGEKGEKLPSPAYHIPGKKNGRYIYATKKKKRDYLSPLGRIFLQDKGNEHID-- 675

PfCRMP3 --------YTYMKLQEE----NGVDLGKNMEGTSELISNILKTN-KLLEKADTIKRMD-- 558

PbCRMP3 -------LNSAQRNRIRNGNFKNKMHDKNNKNFKKKLANFGITN--LLQLNKFIHSVEDY 661

. : . .: .: . . :: . . :

PvCRMP3 YLINGESYLITHLRDLYKVRRYKRNEAKLKGLLSKSVTMVYPAGIVVEKEKVYFVDTALH 632

PkCRMP3 YLMNGENYLIGYPEHLYKIRKYKRHRERLRGLLPNSVTLIYPAGIVVDQEKVYFVDTALH 735

PfCRMP3 EFNNGESYLIKNTKLLNSLRNFPKINKYPISLLNENFTLNYPSGIVVVNDTIYFVDTGLH 618

PbCRMP3 EILNGISYTIGNTPYEEVLSNMPTENMHSQALINDRISLVYPAGIVVFNNKVYFVDTILH 721

: ** .* * : . . .*: . .:: **:**** ::.:***** **

PvCRMP3 MLFCFSLKENRIVETFGYLNAPVMSDKGLNKPFSLALFHVTNWKKSLLFLSELSSSRILI 692

PkCRMP3 MLFCFSLKENKIIETFGYLNSPVMSDKGLNKPFSLSLFHVTKWKKSLLFLSELSSSIILI 795

PfCRMP3 VLFAYSLQKGIITEMFGYVNHEVKSENGLNKPYSLSLFSTNNGNTNLLFLSELTSSRILI 678

PbCRMP3 TLFCYCLIKKEMIEMYGYLNSPIKSDSGLDQPYSLSLYYYDEAKTNLLFLSELSTPRILI 781

**.:.* : : * :**:* : *:.**::*:**:*: : :..*******::. ***

PvCRMP3 FEINKSIKLYLTYS-DLPLDIATSIVVTPQFLIVCGLKRSKENYVNYVTYVKIEELCEFE 751

PkCRMP3 FEINENMQLYLTYG-NLPLDIATSIVVTTKFLIVCGLKKSKENYVNYVTYIKIEDLSEFE 854

PfCRMP3 FEINNSIKLYLIYNNNINFDIITSIVTTSYFLIICGLKQNMDIYKSYITFIKIEELSDID 738

PbCRMP3 FEIDNNIKLYQVYN-DLPFNIATNVITTSRFLVVCGINLNNESITTYITFIEIQDLSDIK 840

***::.::** *. :: ::* *.::.*. **::**:: . : .*:*:::*::*.::.

PvCRMP3 IAYNEFEYALHYGQMVNMTPLKMSKNIKKFTLRQYE-NKTNQNIDTGLNINKHSGMISGK 810

PkCRMP3 IEYNKFPYTLHYGKVVNMTPLKKSSNIKKFTLRQYE-NKSTKNIETGLHIDKHSGIISGK 913

PfCRMP3 IQYNEYDTTLYYGKKFSLDPLIKSPNINEFKMEQID-KFTNKIINTGLTIHKDTGIISGR 797

PbCRMP3 IKYNGFPRSFKYKEVVNIQPLIKSDNIIKFKMNQYDHNIPDNNIITGLKIKKYTGIIYGT 900

* ** : :: * : ..: ** * ** :*.:.* : : . : * *** *.* :*:* *

PvCRMP3 VKISAWFHMEIVVYDYFKKITLNFQNFISSCPKG-FFFESKNCVPCPIGHYTTFADRL-K 868

PkCRMP3 VKISAWFHMEIVAYDYFKKNTLSFENFISSCPKG-FFFKKRNCVPCPIGYYTSNAHRL-Q 971

PfCRMP3 INISAEFHINIFVKDYFKKKKLTFVNFVSLCPKG-FNIENNKCIPCPIGLYYSNSKSINQ 856

PbCRMP3 IEISAIFDIEITVYSNFKKKILILKKNISSCPKGHFYSNNNNCELCPIG-YFSKYNRKDE 959

::*** *.::* . . *** * : : :* **** * :..:* **** * : . :

PvCRMP3 CTSCESYKKNSTTSYTGSISRNECLCKAGYYLKDNKRSCVKCPAGYYKDQVGDFKCQSTC 928

PkCRMP3 CLSCENYRKNSTTSYTGSISRNECLCKAGYYLDNTEKLCKKCPAGYYKDQIGDFKCQSTC 1031

PfCRMP3 CIPCENYRKHSTTLYNSAKSKTECLCKPGYFLKD--KRCVKCVAGFYKDQIGNFKCQGTC 914

PbCRMP3 CYSCEDYLKNSITFYPGSDSENECLCKPGYYLRE--NKCVECPEGFYKDVIGNFKCHLTC 1017

* .**.* *:* * * .: *..*****.**:* : . * :* *:*** :*:***: **

PvCRMP3 ENMKASIVEGAASYEELKCACKDGYYTDAQSK-CVLCHLNNYCIYNPNAPIYKADVIPCK 987

PkCRMP3 ENMKRSIVEGAASYEELKCACKDGYYTDAQSK-CISCYLSSYCIYNPNVPIYKADIIPCK 1090

PfCRMP3 ENTKMSIEVGAKNYEELKCTCKDGYFTEKKKKECIPCPFGNYCIYNPKV-KYKADIIRCK 973

PbCRMP3 EPVKKSTVTGAKSYEELNCSCKDGYYTIYDSV-CEPCPPHNYCLYNPKNKFHKADIILCG 1076

* * * ** .****:*:*****:* .. * * .**:***: :***:* *

PvCRMP3 EHMVTLKRGSTSPKECICSVGYYYEKETDTCKPCSYDTYKPNQGNEECTPFVTNPVSTQE 1047

PkCRMP3 KYMVTLKRGSDSPKDCICSIGYFYENETDTCKLCAYDTYKPNPGNESCTPFVTNPGSTQE 1150

PfCRMP3 NNKLTLHRGSKSPNDCLCSQGYYHDYKMDECELCNYDQYKSHISNDPCVPFISKSLVNQE 1033

PbCRMP3 SKSVTLTNGASSPTQCVCDRGYYYDIMSNSCHPCNFNRYKTTISNEPCEYFVNNPVHSQE 1136

. :** .*: **.:*:*. **::: : *. * :: **. .*: * *:.:. .**

PvCRMP3 FLPNENYIEHSNVFLRKTSNVIISPKKGNSSFRSANFCETGYFYSKNKSICSICRYNGYC 1107

PkCRMP3 FLPNENYFEHSNVFLKKTSNVIISPKKGNSSFHSAKYCETGYFYSKNKSICSICRYNGYC 1210

PfCRMP3 YLINEPYFQYTNIYIKQTSNIIFAPKKGSKMFHEATLCETGYSYSMNKSICSICRYNNYC 1093

PbCRMP3 YLPNENYIQGHNIHLKQTNNSMFAMTKGSKMPQMANLCESGYFYSMNKSFCSICRYDHIC 1196

:* ** *:: *:.:::*.* ::: .**.. : *. **:** ** ***:******: *

PvCRMP3 KGLHNAVTVCPKNSVTVKLKSVTSLDCLCEKGYGRITINKHKLFNILCVPCPYNTFQPHH 1167

PkCRMP3 KGLHNEVTMCPKNSITVQLKSVSALDCLCEKGYGRITIQKHKSFNILCVPCPYNTFQPHH 1270

PfCRMP3 LGLDHSSIICPKNSITIKLKSVSALDCLCIKGYGRIIINTNQSFTISCIPCPYNTFQPHH 1153

PbCRMP3 KGAYNKITQCPIHSVTTKLKSDSYLDCLCARGYGRIIVNDFNNFSISCKPCPYGTFQPHL 1256

* : ** :*:* :*** : ***** :***** :: : *.* * ****.*****

PvCRMP3 SSGECIPCPAYTFTISVRSTSITDCLPQNGYYNMFFQYIYHHSMERLVQNVPRFLQQYRH 1227

PkCRMP3 SSGECIPCPPYTFTISPMSTSITDCLPQNGYYNMYFQYIYQHSKERLVRNVPLFFQQYRR 1330

PfCRMP3 SYGKCIPCPPHTFTKITGATSITECIPKMGYYNISFEYIYEYSKQQMLNSIPHFIQYYHY 1213

PbCRMP3 SAGECIPCPPYTFTKNTKSTSITDCIPQNGYYNQYFEYIYEYTKNKLAQNIPNFLQEYHL 1316

* *:*****.:*** :****:*:*: **** *:***.:: ::: ..:* *:* *:

PvCRMP3 YLSGQYREGREAHLRGGQPSGGSHIDHVCEVPNEDSPLSLHHLTRK-------------M 1274

PkCRMP3 YVNDQYRKGREAHLPKGKPKAILHGEHMCRIPHKHNPLSLHHLTNN-------------L 1377

PfCRMP3 FLKNQY-----EHFAKGK-------NNQKKMNNNNN----NNKKYT-------------C 1244

PbCRMP3 YINDQYAEIKRNVAEKDKNKNNLKKNSGSKTYCYNSDISFDLRGNKYEYYDTKEPSDYFK 1376

::..** .: : . .. . .

PvCRMP3 TRGGKTHGDHEED------HHNAQWKRYDKPHSTSGYKKDLAKARMPKISNSIRNLKGTY 1328

PkCRMP3 MEGGRIHWGHEEN------EKNSQCNWGNNPYATTAYEKDLTKNGIPKISNSIRNLKGTY 1431

PfCRMP3 TTHKRIAINNKSN------KHK-KFPRYINVEEKKYIYKD--KYNIPQITNNIHGLKSPY 1295

PbCRMP3 TYGQKYNKTNAKDSMICSKAYNNDGGNYIEESNFSHAQNNLKKLNINKINNNIRHLKSPY 1436

: : .: : . : . :: * : :*.*.*: **..*

PvCRMP3 SNAFYTNYLKNV-ILMDSSKMGRFIQQEGS-------IFSLAN-SPAGEVYPGGIPKDQR 1379

PkCRMP3 SNAFYTNYFKNV-ILMDTSKMARFIQQEGS-------IFSLDN-SPVEENNPKRMLNDQS 1482

PfCRMP3 NNIFHTNYLKNIFIFMDLFKMNKYIYNQKSKQHRETIIYGHNNLSFVHIKNEKDFSYDEK 1355

PbCRMP3 NNIFHTNYLKNI-ILMDRIKHYNFFQQNPD-------FFKLNN----TERDNIHFYINHN 1484

.* *:***:**: *:** * .:: :: . :: * : :.

PvCRMP3 E----PRQEDTKGAPPKGESI---------LMKDGTEHSPVVPTAEEAASNAPDDRNSYL 1426

PkCRMP3 E----LLQESTKWASSKRESV---------LMKDGKDDLPEITSAEGESSGTPGDSNSNL 1529

PfCRMP3 KSKYNIKMENNNNNNNNNNNINIYSNNNLNFVRQMNEKNKEYTKLLSTKKRNISQSTSFL 1415

PbCRMP3 N----KMGRDKKNTDFSRQSIGFKNVGIRYLLFDKNIKRNNKKNTKINRKNYVGDTIEMI 1540

: ...: . :.: :: : . . . . .: . :

PvCRMP3 K---GKALL----------SEEMKADVRK---FNFIAEKKNDILTMIKTREETYIENRRK 1470

PkCRMP3 N---ESILL----------LEEVKAVARK---FNFISQKRKDLLMLIKSREETYIENKGK 1573

PfCRMP3 N---NKIIYDDYRNLIFNTSDTYNHNVSKS--INIISHDINDILKIIKAREENYFINKNK 1470

PbCRMP3 NNHKNDIRLIG----VTKISSLITGIEKTINEIDILSTTHVDILKTIRTRERIYNSSIRK 1596

: . . . . ::::: *:* *::**. * . *

PvCRMP3 DISYECYEMERAIIIQKN---VYVSTIPENDLISCLNTCISNIYCTGIEMDRTVGHGKTD 1527

PkCRMP3 DISYECYEMERAIVIQKN---VYVSIIPEIDLISCLNRCISNIYCTGIEMDRTIPQGKTD 1630

PfCRMP3 DIKYTCYENEENETSKKRNHFMYVYTIPEIDVMSCLNSCISNIYCTGIEMNTTDDKGKTN 1530

PbCRMP3 KIIYSCYETDQLIKHTRN--TIYAITIPEIDIINCLNKCISNVYCTGIEIDKRHNKWKSH 1654

.* * *** :. :. :*. *** *::.*** ****:******:: : *:.

PvCRMP3 LLFFLNRSDGTKMLHFFKCHLYFYEEIESYYKKE--NLK------RVEDIQQYDDAD-RI 1578

PkCRMP3 LFFFLNKRHGTKILHFFKCYLYFYEEISSYYKKE--TLK------RIEDIQYYDDAN-TI 1681

PfCRMP3 LFLMLYRSYGKTFIYFYKCSLYFYEDLTSYYKKE--NFK------VIKNVEQYNNTHNKI 1582

PbCRMP3 ISYVLTRSSGFENINIYKCNLFLYEDSDSYYDVDDKNINGTKINNKINDIHKYNGTN-KI 1713

: .* : * : ::** *::**: ***. : .:: ::::. *:.:. *

PvCRMP3 TGCIVQKNEMLKLWKVYNFDICPNNYYCPEKSFKKKKCPLNSVKKNFQGTIEDCLCSPGY 1638

PkCRMP3 TACVVQKNEMLELWKIYNLDICPNNYYCPEKSFRKKKCPLNSVKKNFQGTIKDCLCSPGY 1741

PfCRMP3 ISCVIQKNPILHLWKIYSFQKCPHNYYCEQESLKKKKCPPNSIKTIYDGTINDCLCLPGY 1642

PbCRMP3 TRCIIQKNYIFQLWKIYTFEKCPNNYYCLGNSYKKSKCPLNSVKTKFKGKIKNCLCLPGF 1773

*::*** ::.***:*.:: **:**** :* :*.*** **:*. :.*.*::*** **:

PvCRMP3 SLQKNLNLCVPCEKGTYKDSTSNGKCVKCPLNLTTSSEASSSQYDCVCREGYYFQGRFSM 1698

PkCRMP3 SLQKNLNLCVACEKGTYKDSTSNGKCTKCPLNLTTSSEASSSKYDCVCREGYYFHGRFSI 1801

PfCRMP3 YLKGNIQTCIPCEKGSYKNTISNDSCIKCPINFTTLFETSKSVYDCVCRKGYYFSWNTNI 1702

PbCRMP3 YLNVNTKKCVRCPKGTYKYNTSNENCLECPLNLTTISEMSTSIYDCVCREGYYFQGANQI 1833

*: * : *: * **:** . ** .* :**:*:** * *.* ******:**** .:

PvCRMP3 KLVDLKMERAMIEGIKKTKENKISSLPSGQNKVEGLFFQVKSPRR-RQKF--AKMVQPLL 1755

PkCRMP3 KLVDLKMEKVMIEGIKKTKKNKILSLRNGKNKMEDLSFQVKSAGR-RQKF--HKMVQAIL 1858

PfCRMP3 DTNDIEKKNIYNNNINSIYRNKN-EIKLIKELEEEYAFNVEKKRKNKHKFILHKKLNNRY 1761

PbCRMP3 ELINTN--KIKRNILKSYKPGKHSPIQQIKDR----FLEVNQTNN---FFKNSKGIKSKF 1884

. : : . : ::. .* : :: ::*:. . * * ::

PvCRMP3 GE--SPQGTTRER-------EPPRGDNTSVIEENDVISTL----------ERSFTQMGDT 1796

PkCRMP3 GE--SPQGEGGKR-------RHDGGDNTSVNNQNEVASIAK---------ERSLSQMGDI 1900

PfCRMP3 RENIHKKQISLKRKIKFLNEQKEINNHLNFLHVNEILNISFFNDNQNYIYKNKNKQENDT 1821

PbCRMP3 TEQEKVKYTTKKL-------KSKYVAKNNLISINDINFIQ----------SENIQVINLN 1927

* : : . : .. *:: ... .

PvCRMP3 PVHVSQTPHAVQKHYNALP----SVDFLSN--KSDDRVDGLLY------GTCIKCPDKMF 1844

PkCRMP3 PVHVSQSPHAVQRHYNAPS----SLNFLSK--KLYDNVDNSPY------GTCIKCPDKMF 1948

PfCRMP3 NIEINPVSKQTSLHSNNISNKYMRNNFLQINEKLYINNSNNNYNEYIHKAQCTKCPRKMF 1881

PbCRMP3 PIYQNTRNNEKYLHSTVDS-----KGTKKN---IYHNIT----------EQCAKCPNEMF 1969

: . : * . . . . . * *** :**

PvCRMP3 CPGFWYTNFERELHHPPIFCPKGSVVPKTTLESTDVHKCICGKGYSIN----------VQ 1894

PkCRMP3 CPGFWFKNFERELHHPPIFCPKGSTIPKTTLESTDMHKCICGKGYSIN----------VK 1998

PfCRMP3 CPGYWLENYENEIHHPPIYCPKGSLILGTTIESTDIHKCICRKGYSINLSIKPNRNKNVN 1941

PbCRMP3 CPGAWLKNFEFQIHHPPIFCPEGSFIPKTTIISTDINKCLCKKGYGIN------------ 2017

*** * *:* ::*****:**:** : **: ***::**:* ***.**

PvCRMP3 NGHHIRGSDGSDGSDGSDGSGGN----GSSSEMCVKCQERYYKDVVDNSPCAGLCMEFST 1950

PkCRMP3 KNHYNSG--GNNVNDRSDKNGRW----GSSGEMCVKCEEGYYKDIVDNSPCAGLCMEFST 2052

PfCRMP3 VNDNDNGNNNNNSNNNNNNSNGNSATLNNNNNMCVMCKEGYYKDVIDNSVCAGLCIEFST 2001

PbCRMP3 ------------FNKNKNK--------------CIKCKEKYYKDVVDDSLCAGLCMEYST 2051

.. .: *: *:* ****::*:* *****:*:**

PvCRMP3 SFQGSISKKQCFCARGKYMIHESSHEMKCVNCSKGALCIGGLKYKSLKNLIQDSSYTDIE 2010

PkCRMP3 SFQGSISKKQCFCSSGKYMIHESSHEMKCVNCSKGALCIGGLKYKSLKNLIKDSNYTDIE 2112

PfCRMP3 SFKGSISKKQCFCTVGKYMIQDEKDENKCINCSKGSICIGGFKFKSLRELIKNQYYINLN 2061

PbCRMP3 SFKGSISKKQCFCNTRKYMIRESSNEIKCINCPEGSLCIGGLKYKPLKILIKNINYTNIE 2111

**:********** ****::...* **:**.:*::****:*:*.*: **:: * :::

PvCRMP3 IDDHVTPFPQKGYFATFELKHEDFAWSPLNSLNLQINNYDKGDIIIRNKIAKQLFGRRIS 2070

PkCRMP3 INDHVIPFPQKGYFATFEIKHDDFAWSPLNSLNLQINNYEKGDIIIKNKIAKELFGRRAR 2172

PfCRMP3 IYDHSIPLPKKGYFATFQINHEDFKWKPLNSLHIEINNYEKGNIIITDKIILNVFHKKLH 2121

PbCRMP3 VVDHTVPFPQKGYSATFEIVHPDFSWTPLNSVNLKINNYDNSDIVLSNKLLFDVFYTKRN 2171

: ** *:*:*** ***:: * ** *.****::::****::.:*:: :*: ::* :

PvCRMP3 HLTV-ERS--DPNGGITNHKMELKSG----REKKLDYPP--SKESISLVEAGITH---IL 2118

PkCRMP3 YLTV-ERS--DQNDHPKDNKMEIKPG----NEKRLGYAP--SKESNLLVGVGVTH---IL 2220

PfCRMP3 LINIGEKNKYDQADQGIYIKKNLNNNSFIHNNNNIKYIVEESKHLRDRTMANIPNE-PIL 2180

PbCRMP3 YVELEVSKFINGNKTSLLSKNSIFFR------KKIILNISNNKNNKEIYFSKQENKTPVL 2225

: : . : * .: :.: .*. : :*

PvCRMP3 VNEKLEKLKYKNEYLTVERIPDFHLCPISKRCVGGVDNMCAHGSEGYLCNNCSKGYDTIY 2178

PkCRMP3 VNEKLEKLKYKNEYLTVERIPDFHLCPISKRCVGGVDNLCAHGSEGYLCNNCSKNYDTIY 2280

PfCRMP3 VNEKLEPIIYKNNFLMIDRIPDFHLCPISNRCMGTSSNLCFKGSEGYLCNNCSNSYDVIH 2240

PbCRMP3 ITENGEQIKYKKSFLTVDRIPDFHICPIMKKCVGGINNLCYEGSEGYLCSNCSKKYDTKY 2285

:.*: * : **:.:* ::******:*** ::*:* .*:* .*******.***: **. :

PvCRMP3 FRSQCFKCKKTKTELMDLLARKIFFYMIVFFLIYLNYFCYVKRNFVFIGIFKIWYFFIIC 2238

PkCRMP3 FRSQCFKCKKTKTELMNLLARKIFFYMIVFFLIYLNYFCYVKRNFVFMGILKIWYFFIIC 2340

PfCRMP3 FRSQCIKCRKKKTEMINLLIHKFLFYIIIFFIMYLNYYCYIKKNYLFISILKIWYLFIIC 2300

PbCRMP3 FRSQCFKCQKTKIEIFNFILFKAVFYALIFFLIYLNYFCCIKKNFVFIGIFKIWYAFIIS 2345

*****:**:*.* *::::: * .** ::**::****:* :*:*::*:.*:**** ***.

PvCRMP3 FLPYIHIVESNHNSLPNHLFYFQFFITLPMRFLTHYLKLNCFVN-SYSNQQ--------- 2288

PkCRMP3 FLPYIYIVESNHNSLSNHLFYFHFFITLPMRFLTQYLKMNCFMN-SYSNQQ--------- 2390

PfCRMP3 FIPYSFIMEPIENHLKNYQSYFQLYIILPMRFIAHYFKLNCFFNNSYNEINNNITDNKVH 2360

PbCRMP3 FLPYIFIMDSNSPREENYILYSQFFTSLPIKFITQHLKINCFIN-SYNNEK--------- 2395

*:** .*::. *: * ::: **::*:::::*:***.* **.: :

PvCRMP3 -YIHIWYIQRYIKIAEPMIDCAVLTGVFLTIYLVYTRLKGKQISTVEKVIAKQINKVHSR 2347

PkCRMP3 -YIHIWYIQRYIKIAEPMIDCVVLTAIFLTIYLVYTWLKRKKISTVERVISKQINKVHSK 2449

PfCRMP3 IYIYIWYIQRFLKIAEPIFDCILFTLIFFIIYVLYISWNNEKIQIAQSVIKKQIENIQNN 2420

PbCRMP3 -YIYVWYVQRFIKIAEPIIDCFFLVCIFICFYIIYTWWNYQKISLIQKVIKKQINKEYNK 2454

**::**:**::*****::** .:. :*: :*::* : ::*. : ** ***:: ..

PvCRMP3 GYYQHRSDFYYQYYQKHVIDTINDKKAKEDWWSRKWANASFEREQSSCGEAAK------- 2400

PkCRMP3 GYYQHRSDFYYHYYQKHVIDTINNKKGREIFWLRKWENASFEREHSSGEEGGKEQQ---K 2506

PfCRMP3 SYGKHIYDFYYYYYQNNVINIIKNTN--NNCNVKNWSQSYYVHDYLSKTKKKKQKKKLSK 2478

PbCRMP3 EYYKYVSDFYYHYYQKGIIDTINNKRNKTYLEYMKYDCDYFS-SYNSLSFRTPLLK---K 2510

* :: **** ***: :*: *::.. :: : . *

PvCRMP3 ---------------GKVAKKPPH---DSSTMG--------GNTTQCGSSAELS-TSGDS 2433

PkCRMP3 ENTKQGKKKQERNKNGKITKTPQR---SSSTFN--------GNSTQCGSSAHIS-TSDDS 2554

PfCRMP3 WSLTQTKKKLIQRWKHKISRSFKHKKENSENYDYFENKENKENNSLCSESTKHDNTINNN 2538

PbCRMP3 WKNKKCSKKLDKSKLHSVTSWKSNKNQSSKWIN-----QKVSNKSHKWINKKEKKQKNKT 2565

.:: . .*. . *.: . . . ...

PvCRMP3 LRAGKVA---------KKELSSSN--------ENLYNFDEEFLP--RDASNYCLKENIAK 2474

PkCRMP3 FGIRRME---------KKEVSSSN--------ENLHNFNEEFLP--KDESNYSVEENIGK 2595

PfCRMP3 YTTSSVEDK---FIYTKKKTTYNNNTNQSCDDENIYNVKEKLLK--KDITNNFGYEHIEK 2593

PbCRMP3 ISLDDINFLESSSFSRNKELTKENS--KEFDFLDVINFKKDISHKIKKTSSLPIEENKTK 2623

: :*: : .* :: *..:.: :. :. *: *

PvCRMP3 DKYWTYMCALNIYNIRAFGLFRYIHPSSISTFSRLKRILSDLKVLYIIVLYIYFPFTLIS 2534

PkCRMP3 DKYWTYMCALNIYNIKAFGLFRYIHPSSISTFSRIKRVLSDLKILYIVILYIYFPFTLIS 2655

PfCRMP3 EKYWTSICLSNIYNIRALGLFRYIHPYNINIWDKMKRILSDLSCVYIIILYIYFPFIIIN 2653

PbCRMP3 DNYWTSICLLNIYNIKAMGIFRYIHPPNISIHKKIGSILSDLNAIYIIVFYIHFPFTLMS 2683

::*** :* *****:*:*:****** .*. .:: :****. :**:::**:*** ::.

PvCRMP3 LLELVWCQPVKYKNKAPILILYHMPSQVCNWKNQLFASGVIFSSVFFLMYLLLFILSFYG 2594

PkCRMP3 LLELVWCQPVKYKNKTPILILYHMPSQVCNWRNKLFATGVIYSSVFFLMYLFLFIFSFYG 2715

PfCRMP3 LLELVWCQPIQYKENPSLLILYHMPSQVCDLQNKLFLSGVLYSCFFLIIYIILFSIYIYD 2713

PbCRMP3 ILELIWCQSTKYKNKLPILILYHMPSQVCSFQNKLFLSGVLFSGFFFFIYLFLFIKYFYG 2743

:***:***. :**:: .:***********. :*:** :**::* .*:::*::** :*.

PvCRMP3 TLKNFKIFGSYAKRVRSYFLFNGYNYQNRCWDLFNVVKVMFVAISFTCQLYTKRSHNAKY 2654

PkCRMP3 TLKNFKIYGSYSKRVRSYFLFNGYNYQNRCWDLFNVVKVIFVAISFTCQLYTKKIHNAKY 2775

PfCRMP3 TSKSIKAIGSYSNNLKSYFLFNGHNYQNRCWEIINIIKVSFIIIPFICQLYTKRNVNSKY 2773

PbCRMP3 TFKNFKVFQSYDRDFKCYFLFNGYNYQNRCWDFVNIIKIVLFVTSFMCQFYTNKINNSKY 2803

* *.:* ** . .:.******:*******::.*::*: :. .* **:**:: *:**

PvCRMP3 FICCCIVFILITEVTLILMYSPYDKRSNNILRKLSLLSTFSILITYLSVQFSFFFNFPIT 2714

PkCRMP3 FICCCIVFILITEVTLILMYSPYDKRSNNILRKLSLLSTFSILITYLSVQFSFFFNLPIM 2835

PfCRMP3 FICCCIVLVLITEVACILFYSPYDKRSDNLLQKLSLTSSFFILISYLTVQVGFFFDVTIL 2833

PbCRMP3 FIFSCIIFIIITEITLILLYSPYDKRSNNVLQKLSLLSIFSVLITYLTTHFSFFFDFYII 2863

** .**::::***:: **:********:*:*:**** * * :**:**:.:..***:. *

PvCRMP3 NALPFVLFVYFHLYMGNKIVLEFAIYRNIFKRKEEKEDQQDAGREAEGLLNEQSFFNFA- 2773

PkCRMP3 NSLPFVLFVYFHLYMGNKIVLEFAIYKNIFKRKVEEEDQQDVGREAEGLLNEQSFFNFG- 2894

PfCRMP3 NIIPIFLFFYFHVYVIKQIILDFAIYKNILKRK-DNHISFDKKKKSNDMLNDYNFLFFFD 2892

PbCRMP3 SALPFILFIYFHIHTINKIVLEFALYKNILMKPTRQKQKLD----SEEFLNENSILFNS- 2918

. :*:.**.***:: ::*:*:**:*:**: : :. . * :: :**: .::

PvCRMP3 ------KQKSSQNGVPLGLHAD-------DGDGSVPQNGCSDGGEK--GKKKTSFPFFPL 2818

PkCRMP3 ------KEKSNQNGAYLNAHAD-------DRITSGTHNSSTEREEK--KKKKTSF-FF-- 2936

PfCRMP3 KIEIDNKDSNTPKGIDTTVQCDEKEIKNKDDINNFNHNKINDVNNKRKKKKKKKKKKKKD 2952

PbCRMP3 -------YLNTKTNIQ---DND-------EDIIFSNSKKIS-------RKYKRSK----- 2949

.. .. . * : : . * * .

PvCRMP3 SSPYPSIFRHCNLCDLLLRANNIPFYSILFNEQGEEIFIFEKGPTK-CKY-EEIVKKVKM 2876

PkCRMP3 SFPYSSGLKNCYLYDLLLRVNNIPFYSILFNEKGEEIFIFEKGSTK-SKY-EEIVKNVKM 2994

PfCRMP3 ETTKSPYNEEYNLLNFVLSSNNIPSYSIYFDDKEEEIYLFQNNNTRGYEYKKKIINNMKY 3012

PbCRMP3 KEIHSKIKENYNLLNFVLRINKIPSCSIFFNEKNEEIFVQDSNDLIKCKYYKNEKYFKKH 3009

. . .. * :::* *:** ** *::: ***:: :.. :* :: *

PvCRMP3 N-TTERSQTGVISLD-----LLYYNIRDPSRQS-LSNVN-Y--VDLNKSTTNWSQL---- 2922

PkCRMP3 D-TTDRPHTGVISLDLLHSDLLYYNINDPSRQP-LSNIN-Y--VNLKKTSTNWSQR---- 3045

PfCRMP3 NNDIKIEQTKRISLDLLHSDILYYNIRKGTINSDISNMNTYNNIKMRKQENNYYHQNDCN 3072

PbCRMP3 NNSLKIN-PNIITLDILYSDLLYYNTTY-SNNEIFTNLN-----NSPPPKYCYSSSG--S 3060

: . . *:** :**** : : ::*:* . :

PvCRMP3 -MG---------EGKLSNN--YEKFWKREKEYP----REGDNYPHGEVDKENSRRAIPPI 2966

PkCRMP3 -MG---------ERKLSNN--YEQCLRREKKYS----KEKGNNLHDHEDRENSRRAIPPI 3089

PfCRMP3 NINSDDYNNVCVQKRISNK--LEHFIRIEREYD----KEK-HYIKNN-EKNNSEDDMFFL 3124

PbCRMP3 KMSK--------DESLSNRNGFREFSNTEKIVESSESYEEKNVLDNQIQKDTLKNYNSPL 3112

:. : :**. .. . *: * : ... :::. . :

PvCRMP3 NITHFINCLIEAINILFVNQSYNQISVEWISFVTRFSICFIHWMKNHDENLVNLVPVNKK 3026

PkCRMP3 NVTHFINCLIEAINILFVNQSYNQINVEWISFVTRFSICFIHWMKNHDENLLNLVPINKK 3149

PfCRMP3 NITHFINCFIDAINILYINQSYDKITVEWLSFITRFSICFIHWMKNHDENLINFVPLNDK 3184

PbCRMP3 NKTHFIKCLTDAINILFVNQCYNHISVEWLSFVIRFSICFIHWVKNHDKNIIDLPPLNKR 3172

* ****:*: :*****::**.*::*.***:**: *********:****:*:::: *:*.:

PvCRMP3 QFEMKKRNLLFYSLFSSYAEVYSLHSTIGDNNKFEEFKKNFVQAENLEQFYIYLNEANKK 3086

PkCRMP3 QFERKKRNLLFYSLFSSYAEVYSVRSIIGDMDKFEGKKKNFLQAEKLEQFYIYLDEAKKE 3209

PfCRMP3 QFELKKRNLLFYSLFTSYTEIYNLYHSINNSNSLAGCKKRFINSKNVSKPYKHIKKIKNK 3244

PbCRMP3 QFELKKRNLLFYSLFSSYSELYSLYFHINKYNKFETNKNDFVKAENLEMFYMYLTEVNHL 3232

*** ***********:**:*:*.: *.. :.: *: *::::::. * :: : ::

PvCRMP3 SP--------------GEK--PAYRQSD-------------------------------- 3098

PkCRMP3 TP--------------PAK--PVNSQTN-------------------------------- 3221

PfCRMP3 QGDLYGINNLNDTKDIGDKYQRNYKRNNYYNGNIKNEHLNNYDLNNYDLNNYDLDDYHIY 3304

PbCRMP3 NI--------------QTN--GENTQGD-------------------------------- 3244

: : :

PvCRMP3 ------------------------DEEKSD-------CYTP----LNFLDDEFYRCEQDI 3123

PkCRMP3 ------------------------DEEKSK-------CYTP----LNFLDDEFYRCEKDI 3246

PfCRMP3 NNDIYNNDIYNNHIDNIHLDNNHLDNHKKDKPFIITSASSPSLPCFNISDDQFFRCENDI 3364

PbCRMP3 ------------------------NDKIPE-------YFVP----SNLLDDEFYMCEHDI 3269

::. . * *: **:*: **:**

PvCRMP3 IKLLFDENIFKNLSISIVEFFFSIYMIQFIESKRLSILIFLFCEKKN-YLSREKQFLKMI 3182

PkCRMP3 IKLLFDENIFKNLSISIVEFFFSIYMIQFIESKRLSILIFLFCEKKK-YLSREKQFLKII 3305

PfCRMP3 IKLLFDETLFNNLTITLMEFYFAIYIIQFIESKKLSILINLFCEKKK-FLSKERQFLKIL 3423

PbCRMP3 IAMLFDKSIFRNLSITLLELQYAIYMIQFIESKRLSILIFLFCEKKKKKMIKEKSYINMI 3329

* :***:.:*.**:*:::*: ::**:*******:***** ******: : :*:.:::::

PvCRMP3 EARKKDIQLIMYSQN-NKQMYDFVENNYLNEYNRGLRKKIKKLQNLIRKKEK-AALKQEE 3240

PkCRMP3 EARKKDIQLIMFSQN-NKQMYDFVENNYMNEYNQDLKKKIKKLQNLIRKKEK-SSLKREK 3363

PfCRMP3 MSRKHIIQKIIDKNNLSTHMNEYIEYNYYLEYNKKLKKQREKLLNLIINKKG-QIRTLKS 3482

PbCRMP3 KDTKRHVQNILSNDTNINQLYE-VEHRYLKENNYIIKNKIKALQKIINKKQNNKYPKIDN 3388

*: :* *: .:. .:: : :* .* * * :::: : * ::* :*: . ..

PvCRMP3 VKSVTRVQGNKANKISNANAYFVQTLKKLLDDSSATARRHTGGAKRNPHERAPQ 3294

PkCRMP3 VKSITRAQGNTANKMSNANAYFVQTLKKLLDDSSATTRGHTGGAKRKTQERTPN 3417

PfCRMP3 IKNLQLMEENMVQGKNNINTHFVKTLKKLLEHQPS----HQ--SENNIK----- 3525

PbCRMP3 IKSIMTEE----NGKNKYDK---------------------------------- 3404

:*.: : : .: :

**B) Alignment of PCRMP4 from *P. vivax* (PvCRMP4)*, P. knowlesi* (PkCRMP4)*, P. falciparum* (PfCRMP4) *and P. berghei* (PbCRMP4)*.***

PvCRMP4 MKCEKRGRALNLSLLTIWLVIFLKNIKASIRISDFTYPSEKAVLNAEQIETDYISILKLK 60

PkCRMP4 MKYEKKGQVLNLSFFTLWLVIFLKNINANIRISDFTYPSEKAVLNAEQFETEYISILKLE 60

PbCRMP4 MKFKPNIKNLNAFFFILYFVRLLQNINAKISISDFNYYNDVNDISVSLLETEYISILKII 60

PfCRMP4 MK--------NITFLVS-FLIFLRSINANISITNFTYTNQKGDIKVEFFQTDYIAIFKLP 51

** * :: :: :*:.*:*.* *::*.* .: :... ::*:**:*:*:

PvCRMP4 NDGKSTPNFEISDRYLISEDNTIQNCYAPNDDHGKVLIKKNDKNKFLFVQKQDEDIFIKD 120

PkCRMP4 NDGKAAPDFEITDRYLISKDKTDQNCYAPNDDHGKVLIKKNDKSKFLFVQKQNEEIFIKD 120

PbCRMP4 GDGTTNTNFENSYDYLISDDEQDKTCNVIHNKLGNVIIKKNIDSDIILVQKTNVKTYIKD 120

PfCRMP4 FGGSHEQIFEISDDYIITNDKETNNCNIPNNNFGFVYSKSNSQNKFLIVQREYTTIYIKD 111

.*. ** : *:*:.*: :.* ::. * * *.* ...:::**: :***

PvCRMP4 RNYKWSLISSLEKTDRPYQIVQENHEKITVGDIANCDSNTRKKFIDYFNLLDSFFISENE 180

PkCRMP4 RNYKCSLVSSLEKKDPQQQIVQENYEKINVGELANCDDNNTKKFVDYFNLLDIFFISENE 180

PbCRMP4 KNNLWSTITPIKESIYTIQEIKKEDDHISVGDIEQCNDENIRIFTNYFDILKDIIVSDSE 180

PfCRMP4 KNNNCSLISKVEPSLYSFQTIEYKKEKIKLGTTTKCDEKMVEKLKIYIDLLNDMNLSEDK 171

:* * :: :: . * :: : ::*.:* :*:.: . : *:::*. : :*:.:

PvCRMP4 KCETVLKTPLTKNVQVIDCVGYIGHITNFPELKHSTYYICFKT--------NESSLLIST 232

PkCRMP4 KCETVLKTPLTKSVQVIKCVGYIGHITNFPELKDSTYFICFKT--------NDLTLLIST 232

PbCRMP4 KCNQIIGPTFVKKTKVLNCRGIIGHIKDIHKLYNGTYYICMKD--------NYISLILSK 232

PfCRMP4 VCNQIIASSFIQNIEIIDCTVYIGSIFLRNNLNREEYYICIKKEDNNNKMKNNISLLINT 231

*: :: ..: :. :::.* ** * :* *:**:* * :*::..

PvCRMP4 FSADKTIANNTFYCTFDNPVCPLTISVSLFNNVPYIMDKSLVLKPDCSVISTPIYKEDHV 292

PkCRMP4 FMADKTIANNTFYCTFDNPVCPITIGISLFNNIPDIMDNTLVLKPDCADISTPIYKHNHV 292

PbCRMP4 IIIDKPIPTSTFVCNYRNSNCLIPINANIFSIVPITNDETVILKSKCDDTSSPHTKTNHK 292

PfCRMP4 IDLNNTLIMNTFYCNMERDICSIIINSIYFNNIPNITDDTLVLKAQCNIDSNPLINIDHV 291

: ::.: .** *. . * : *. *. :* *.:::**..* *.* : :*

PvCRMP4 LSGDHYIFFFKNDNTTYYREICILKDMK-YHKVGNLFSIEIYGPIICFIGSECVIKTYTA 351

PkCRMP4 LSEDHYTFFFTNNEKAYYRHICILKDMK-YEKVGILFFIETYIPIICFIGSECVIRAYTA 351

PbCRMP4 VDGEYYVFNFENN-KEYYLEVCIKNMSK-YIIVGYVFFLEAFNPITCFENSQCPIKIYTP 350

PfCRMP4 LREGYYTFHFRNP-KIYFLQICIIDNLKKYRMLGKIYFLKAYEPIICFIGTPCVIKSFTV 350

: :* * * * . *: .:** . * * :* :: :: : ** ** .: * *: :*

PvCRMP4 VEKMQYIMNNIESTTFSWSPSNCNNKDAEKNA--ITTYP----KEEDGLLYIISEKILLQ 405

PkCRMP4 KDKFQYILNNVESTTFSWSRTTCNNRTMEKND--ITTYL----KEEGGYLYIISEKHLIQ 405

PbCRMP4 KDKINYIANNIKLVNLIWSNTECNQNKSTDKK--ISTNI----QKNDDTLYITSADPIQN 404

PfCRMP4 KENVQYVQNNIDNETFIFTNKKCDNENVNENMNMIETDYDMKELENHQHFYLFSKNPLTD 410

::.:*: **:. .: :: . *::. .: * * :: :*: * . : :

PvCRMP4 ENILCTKNNTKLRHLLTIELVENPQYSLYYSIINSVIMNDNWQNIKYNNYLGPFVKYECS 465

PkCRMP4 ENILCIKNNTKLRDLLSIELVDNPQYNIYYSLINSVIKNNPWQNIKYNNYSGPYVKYECS 465

PbCRMP4 ENTLCSNNNEYYLPLFTIHLIKNPEYEIHYTIIKGVVFNTMKYLFEYNNYTGPYIKYECS 464

PfCRMP4 EKYLCTSNNKKFLLLFNIVLIKLPEYNIHYSIINNVVINYSTLFFEYNNYLGPYVKYECS 470

*: ** .** *:.* *:. *:*.::*::*:.*: * ::**** **::*****

PvCRMP4 HEEIVINEYDIWKSNYDNEYIHKNEELNFNIDFS-ALTMRLCVKQIN-YIDIGSVRIEQL 523

PkCRMP4 HEEIAMNEYGILKSNYDNEFIYRNQELNFNIDFN-ALTMRLCVKQIN-YIDIGSVRIEQL 523

PbCRMP4 YNLNVKDEYNNSVDNSDNEFIHSLGKLNFNTNENNAITMRLCLKQYN-YYDIGFVKIEKL 523

PfCRMP4 DKSIVPDEYNNFNNNKDNNFLLKNEELNFTIKFK-AIIMRLCLKQNNYYYDIGKIYIENF 529

: . :**. .* **::: :***. . . *: ****:** * * *** : **::

PvCRMP4 FGFKVNN-LDPLEVSAIVSLYNEDINTRLLKFAIKEDENCNSSKGFFIHSKG--NYPKEI 580

PkCRMP4 FGFKVNN-LDPLEVSAIVSLYNGDINTRLLKFAIKEDDECSSSNGFFIHSRG--NYPKEI 580

PbCRMP4 TNIKNIS-FNPFLFEGSILLYKDNVNTKISRFAIKEHENCHSDKGHFLMSTAYTEYVETL 582

PfCRMP4 INYKKISSLKDFIFNADVSLHADDINTKLLKFAIKEDHNCHSDQGFFVQSKD--NFRADK 587

. * . :. : ... : *: ::**:: :*****..:* *.:*.*: * ::

PvCRMP4 --FHKDIIVDARAEYEVQEEINLDKPRYYLCMCSLAEKCNYSNGLSAYNLYTNIYINNEI 638

PkCRMP4 --FHNNIIVDARAEYQVGKEINLYKPRYYVCMCSLAKNCNERDGLSIYDLYTNIYIDNEI 638

PbCRMP4 N-IHKYVSISAKVNYKIDSQPKLDKSRYYLCMCSDEKLCKNNNNLINYYTYTNIYIDNET 641

PfCRMP4 KPFYKNIFINHTTVYESINQIILDKDIYYICMCSLHEKCYDNNNLIDYNVYTNISIQNEI 647

::: : :. . *: .: * * **:**** : * :.* * **** *:**

PvCRMP4 IPQE-KKKYVCKLFSPCNFTVKFES--DNISNLWAAKVGTSCRT-YAFADIKIKFKKNDK 694

PkCRMP4 IPPE-KKKYVCELFSPCNFTVKFES--DNVSNQWVAKVGTSCRT-YVFADIEIQSRKNDN 694

PbCRMP4 KDLS-KTVYICKLFYKCTFTIQFSS--SQISDKWVSKVGNSCNT-NKIVHIGIDHKYNNV 697

PfCRMP4 QPLQNKKKFICKIFADCSFTINFENENNNTSDIWVAKKGQSCNSKDIQVGIHVSHQNVKE 707

. *. ::*::* *.**::*.. .: *: *.:* * **.: . * :. : .

PvCRMP4 LSKADSNIIAVDFSVFYNVSQIN-NNILNEDLVICGNYKDQKFAFTIEADFFFTYISSPY 753

PkCRMP4 LSNDASNIIAVDFTIFYNVSQMN-SNILNEDLVICGNYKDQKFTFSIKSHLFFTYITSPY 753

PbCRMP4 LTDHKSNIIALDFNIYYNINEWN-KNLFNTNIVVCGNYNNQSFNIYIENDYFLIYHNIPN 756

PfCRMP4 INKEKINVTKVNFKIYYLISHIHYSYLFNKDIFICGNYRNQHFIVHIKSDFFLLYYALPF 767

:.. *: ::*.::* :.. : . ::* ::.:****.:* * . *: . *: * *

PvCRMP4 -QKFEIKNIEYENLCRDK--------------------KTLNIYSYQN-GETNLYKKVAI 791

PkCRMP4 -EKFEIRNIEYENLCRDK--------------------KTLNIYSYQN-GETNLYKKIDI 791

PbCRMP4 -IEFDIINIKHDKLCNNK--------------------KDLYIYSYFN-GQENLFKEIKI 794

PfCRMP4 EEEINIKNVTQTNVATNTYEAQEKNINIQSFENVKQKFKRLFIYSYRDDAKSNLYKTMNI 827

:::* *: ::. :. * * **** : .: **:* : *

PvCRMP4 DINSN--LSEIELNIEYDH-LKYYKIFFIKECHYCKGVITYTHR--GNSYSLHNP----- 841

PkCRMP4 DINNN--SREIELNIEYDH-LKYYKIFFIKECHYCKGVLTYTHD--GSIYSLHKP----- 841

PbCRMP4 DEKTN--HNQINLKIDYND-LKNNQLYFIRECHYCPNVLMYHHN--DDHYIFQNT----- 844

PfCRMP4 NQDENQISNTIKFEIPNDHPTKYNEIYFINECHYDQGVIIYDHKRESNNIAINNDNNNNN 887

: . * *:::* :. * :::**.**** .*: * * .. :::

PvCRMP4 ----VRNPEKYWEPK--IGKMNRNNK-DFKKSIKMHNCAYETMQNHALTSLLLFDGPHEE 894

PkCRMP4 ----IVNTEKFWEPK--IGKKNDNNR-DPKKSIDLHNCAYETMQNHTLTSLLLFDGPHEE 894

PbCRMP4 ----VNMPERNFDQM--IYENNLISL-NMDGSINIYNCESSTKENYIVTSVIMFDGPVQE 897

PfCRMP4 NNHIIKSKELYWARKKRIYNKKLKKKENKNEIINIYNCSNEIMNNHTLTSILIFDGPLKP 947

: * : * : : . : . *.::** . :*: :**:::**** :

PvCRMP4 INFYCYRGTNCRHKAKFTLLWYHYTIYVELTETERIIISYRE----NKEKKIIAEEILLG 950

PkCRMP4 INFYCYRGTNCLHKEKFTLLWYHYTIYAEITETERIMISYTE----IKTNRSTASGILSG 950

PbCRMP4 INFYCPINTDCKHTEQFYLLWYKYSLYIEVTDTKRIIVVLHE----IPEKKLNHPNSFST 953

PfCRMP4 LDIYCYMELDCIHKENFSILNFDYSLFVEISDTQRIIIHIKESEGNNKEKLNKAEKIYFN 1007

:::** :* *. :* :* :.*::: *:::*:**:: * :

PvCRMP4 --MRNSDLYLRISKHVIEKMNPGPYKIIYSRKIGESFKENYIGTFYYVGPSRQPFQIIAK 1008

PkCRMP4 --IRNSELHLTISKHIIDKINPGPYKIIYSRKIGESFKENYIGTLHYVGASRQLFQITAP 1008

PbCRMP4 --IKISKMILIVSSIKTTEMKPGPYKLIYSKNVNGVIVESYVGTFHYIGPIVH-KNIVSD 1010

PfCRMP4 DSKRTTELILSIPKHISKGLEPGPYKIIYTRKFKQFIHELYVGTFHFIGAPSQKYHLIRE 1067

: :.: * :.. ::*****:**:::. : * *:**::::*. : ::

PvCRMP4 DETEITLHGYFKNINDKHITVFRYL-TCDFYTFLRNNYRELWDLEKIYDNIKEEIFNNIQ 1067

PkCRMP4 DETEITLDGYFKNILQEQITVIKYS-TCDFYTFIRDNYGRLGNLKNIYDNILDEISSNIQ 1067

PbCRMP4 VANNILLEGYFKQIENDKIKIIAYS-TCNFTKLLTD----ISKPNENVNEIKKTILSNTH 1065

PfCRMP4 NQENIIIHGYFKNIENERIHILKHQNTCVLTN--KDKNKKLLKSSE-IEYILNEIKTNIQ 1124

:* :.****:* :.:* :: : ** : . : : . .: : * . * .* :

PvCRMP4 SCTVSGEDKLTCKDMEPIRKKYALCWCY---GDRQNFCNRLENMQFLITEISSLDHSINP 1124

PkCRMP4 DCIVSSEKKLTCKNMKPINKKYSLCWCY---GDRKNYCNLLENVQFLITEVASLDHFIKP 1124

PbCRMP4 ECKYNNDHTLICEDAQLNNEKINLCWCY---EISEKYCENLLNVQLKIIEYSPLQSILNP 1122

PfCRMP4 NCTYLDDTKLLCKNDLPVEGKFILCWCYNILNDQNNLCHNLNIYRPKITEVSSLLYSIDP 1184

.* .: .* *:: . * ***** :: *. * : * * :.* :.*

PvCRMP4 LVQINMPPNAVFMNYNTEGYFFLVEDDCHDIKHFHLKSQLNKEEHILNLYKELESPKKYN 1184

PkCRMP4 IVQINKPPSTVFMNFNTEGYFFLVEDDCLDIKHFHLKSQLNKEQDILNLYKELESPKKYN 1184

PbCRMP4 ITQISKSSNFNFMNYKTDGYFFLIEKNCLDIKNLDLKSNLSDPNHIQEIYKRLKYPNKYN 1182

PfCRMP4 ILEISKSSLTNFLNYNIGDFFFIIDDDCKNIKNIKLKSKLNDEQNILDIYKHLQYPKKYD 1244

: :*. .. *:*:: .:**:::.:* :**::.***:*.. :.* ::**.*: *:**:

PvCRMP4 LCVCKVTQNVACDSENQFKPLELEFGYTIDDKEKLKDDLQH-FNTGMRAVKDFTFSSEFM 1243

PkCRMP4 LCVCKVTENVACDSENHFKPLELKFDHTLYDKEKLKQDLQN-FNTKFTAVKDFTFSPEFK 1243

PbCRMP4 MCVCILKENILCDKTTDFSRLIVTIEPISY--KLIKQNVNDDFNKNSNKVHDFDFDPDFK 1240

PfCRMP4 LCICIVQHNIPCDKEENFKNFYIKLNEQIY--FTMEMGSIDEFNNKDKFIKDFTFSTDYL 1302

:*:* : .*: **. .*. : : : :: . . **. ::** *..::

PvCRMP4 ELLHKGVMQAMVFLSGFMGYKEYVCLSGVPCKTPIKMQTVYSSQVKY------------- 1290

PkCRMP4 QLLDQGVIQAMVFLTDFTAYKEYVCLSGIPCDASIKMQTVFNSEMKN------------- 1290

PbCRMP4 EKLDEGVAPVTIILPGPIQYNNYICFSGILCRT--------------------------- 1273

PfCRMP4 DQLKLNIVPVFVFLPGPVEYKEYVCFAGIPCNATIKIKRSAQTRLITGNKQIIGEKKDVA 1362

: *. .: . ::*.. *::*:*::*: * :

PvCRMP4 --------FLDIMQLKKMKQIVKKESTDKER-----------------------ENK--- 1316

PkCRMP4 --------FLHLRELKKMEQIPKEDGTAKKE-----------------------ENKEIK 1319

PbCRMP4 -----------IINFKRIIPISELYTSNKRI-----------------------EDK--- 1296

PfCRMP4 NPYTGSYGNRNKANIKKGEEKKEQKRNDKKEGEIGYKKEGEIGYKKEGEIGHKKEDELGD 1422

::*: : . *. *::

PvCRMP4 ------------------------------------------------------------

PkCRMP4 DEE------KEEKKEEKKEEKKEEKKEEVKEEKKEEKKEKKKEEKKEKKKEEKKEKKKEE 1373

PbCRMP4 ------------------------------------------------------------

PfCRMP4 KKEDELGDKKEDNNEDQNQDNKQDKIENMNKDINKDKEIKTKETNIEEEEEVEEEEVEEE 1482

PvCRMP4 -KGQAQEIE--EEEEEEK--NKENE---------------------------------DP 1338

PkCRMP4 EKGEKKEEEKGEEKEEEKEEEKEEE---------------------------------NP 1400

PbCRMP4 --GKPKKAN---DDNNKR------------------------------------------ 1309

PfCRMP4 EEEEEEEEEEEEEEEEEKEEEEEEEQEEQFDHNEHEVKGADKNKDKNFNQNKGESQNVNP 1542

: :: : :.::::

PvCRMP4 LMYFS-YLRPHPDDTET---------KEDTIYFTLSDTRECKNEYSQFYKEVKGEIVRTQ 1388

PkCRMP4 LMYYSSYLKAHIDTTET---------TIDTIYFTLNDTKECKNEYNQIYKKAKGKIIRIQ 1451

PbCRMP4 -------IAKHVKEVG-------------ELYFTLNDTKECKNENQKIYKSDLGDVIIAQ 1349

PfCRMP4 NTQQNQYDEQIISEEEQDNIENSLLGKHQYIYYTLNDTKECKNEYETVYKSNASSIYVSQ 1602

. :*:**.**:***** . .**. ..: *

PvCRMP4 EIDLTLQKILEVSFDLNIDFIPPDVRKPYEICAFLRPTDKHYYNIGNVIFYGLFNE---- 1444

PkCRMP4 EIDLTLQQILDVSFDLNIDFVPPEVNKPYEICAFLRPTDKYYYNIGNVTFHGLVNE---- 1507

PbCRMP4 EIQYLMYQLVDVIYDLKINIVPQKINKIYEICASLSLKDINYYNIGSVTFYGIFNENYNP 1409

PfCRMP4 EINYLHHEFIDGVYNLKIDIVPTDITKRYEICASFYEDDKYFYNIGNIIFYGIFNE---- 1658

**: :::: ::*:*:::* .: * ***** : * :****.: *:*:.**

PvCRMP4 ------------SKLVEIVSGIPFNLQLKQFNSKYNVRIRFVYEHYEDSNACKNNYGNSE 1492

PkCRMP4 ------------SKVVEIVSGVPFNLELKQINSKYNVKIRFVYEHYEDSDVCKNNHGNSE 1555

PbCRMP4 GNIKSAEDGELGSNYYEIVSGVSFTLEVKQFYTSYDIFIRIVYQNFGDGPFCHINNNTRE 1469

PfCRMP4 ------------SNTLDIISGVPFSFEINQFYSKYDLFIRLIYEKHGDSKTCRLYNNNFE 1706

*: :*:**:.*.::::*: :.*:: **::*::. *. *: .. *

PvCRMP4 NFFYSESVQQLLTADEKEEIGGKVTVHKWNDLLTVLDADNYNIMVGHRNDDGSHNLHVCS 1552

PkCRMP4 NIFHSDSVQQLLTPDGKEEIGDQVTIHMWKDLLTILDADNYSIMTAHRNDDGSNNLQVCS 1615

PbCRMP4 KIYSRNTVQEFLLQHEKKNIADNVTSHKWEDLILSLDVDNTNYMASHKNIDKPQYVLVCS 1529

PfCRMP4 NVFLEGTIERTIVQHDKKVLDNYITNYKWKDVTIILDTDNNNYMTSYRNVDGSYNLYVCS 1766

:.: :::. : . *: : . :* : *:*: **.** . *..::* * . : ***

PvCRMP4 CYELVEGLCNNDYYYSADSMIIKVHTAVLTEPPMNSIIHFMKPFTLKLKTSKVLNGSIRI 1612

PkCRMP4 CYELVDGHCDNDYYYFANSMKIKVHTALLTEPNMNSIIHFMKPFTLKLKTSKVLNGSIRI 1675

PbCRMP4 CYELIEGNCRSHDMYTSEIMKYRMHTAILKSPKINSTLNFLEPVTITLQTAKELSGSIKI 1589

PfCRMP4 CYELTEGHCQSGNYFSTQVMKFYVHTVELKEPKMNSLMHFLKPFTLKLETTKILNGSIKV 1826

**** :* * . : :: * :**. *..* :** ::*::*.*:.*:*:* *.***::

PvCRMP4 FPIDIMKDLSKICLEINQRDYFLTFP-AKVEEYEQTYDANVNFLSQAVVACWCSKEKCES 1671

PkCRMP4 FPIDIMEDLSKLCLEINERDYFLTVP-AKVEEYEQTYNVVINFPAQGIIACWCSKVKCEH 1734

PbCRMP4 FPIEMEEDLNKLCLELNRRKMFIIYDKSDLNEYEQNYEIIIDFLAQGIIICWCPKHMCDD 1649

PfCRMP4 IPIDIISDLSKLCLELNLKNIYLKYQ-AEINEYEQTYNITINFLSQGFIICWCGKDNCKD 1885

:**:: .**.*:***:* :. :: :.::****.*: ::* :*..: *** * *.

PvCRMP4 ADYINKVAFFKLSSPSILEVHTDMDGYFTFSFLNEWVHINDRILFVAEDLPCTDDTSASL 1731

PkCRMP4 DDYLNKVAFFKLSSPSPLEVHTDMNGYFSFSFLNEWIHVNDRIWFVAENLPCTDDKSANL 1794

PbCRMP4 NDYLTKVAFFKTNAPLFLKVDTDPTGHFSFNFLNNLISIDDRIIFVDSNFSCTDNVHDNL 1709

PfCRMP4 HEYLTKVAFFRLNSPNFIDVHTDLNGNFSFHFLNQFVNINDRVRFVGQASSCLDDENVDL 1945

:*:.*****: .:* :.*.** * *:* ***: : ::**: ** . .* *: .*

PvCRMP4 FDDIIHIN---NKE---------------------------------------------- 1742

PkCRMP4 YNDIIYIN---NKK---------------------------------------------- 1805

PbCRMP4 FDNTIEID---NQT---------------------------------------------- 1720

PfCRMP4 FNNHIVIKNVQNKKGKTSNGGNTKKEQQKNNNKINSNNNEEDENDNENKNERKDEDDNYE 2005

::: * *. *:

PvCRMP4 -YHIGWEQIIEIYGKPRKIVKEIDDNFTWISKIYKITNILEKHLKICYCFYYYSENCKDR 1801

PkCRMP4 -YHLHWEQSIEIYGKPRKIVKEINDNFTWISKIYKITNILEKYIKICYCFYYYNENCKDQ 1864

PbCRMP4 -YSLSFEQKLNIYGTPQKIVKEINGNFMWVSKIYKIK-KPEISVKICYCFYFYNNNCNDV 1778

PfCRMP4 SYSIDWEQIIEIFGKPRNIFKGINQNWTWVSENYKMKTSNEKYIKICYCFHFYYKNCKDT 2065

* : :** ::*:*.*::*.* *: *: *:*: **:. * :******::* :**:*

PvCRMP4 KNYSYIGLLHNHTLGKGVADKNDQIDQ---TVNMYTLNGNSIKFVPMISYNDSNRDICNT 1858

PkCRMP4 KNYTHIGFVHNHTLRKELTDENEQIDQ---TINMYVLNGNSIKFVPMNSYNDSNRDICNT 1921

PbCRMP4 NSYIYIGLVYNNTLMEKNITNSDLIYK---SPNINYFSKDSIKFVSINSFN-KYANVCNN 1834

PfCRMP4 KNFLYIGLVHNHIWDSKTTIMQNELDTNYEISNMIYFNKDSIKFVAINSFNDLNRNVCDN 2125

:.: :**:::*: . .: : *: :. :*****.: *:* ::*:.

PvCRMP4 RGNQSNGIIPIFKN-TAFNNSANFKLRILNIFKQHYQNEINFVVCYSSYYYRYKNTYLLR 1917

PkCRMP4 GRNQSNSIIPIFKN-TALN-YINFKLRILNIFKKHYKNEINFVVCYSSYYYGYKNTYLLR 1979

PbCRMP4 RTHNNFVIIPFVKN-EAFQ-YHDYKSRILN------------------------------ 1862

PfCRMP4 INYEKNIIMPFFSGYNKFHNTTINQKNILNILKTYYHNEFIFILCYNTEYYKKKTNYLLT 2185

:. *:*:... :: : .***

PvCRMP4 KVEYNEL---SRAPNPIIHNYTLGHAGITSES-SQNLVPRREHREKIENLLKSYSKIKQR 1973

PkCRMP4 KVEYSEL---SRAPNPTTHNYMLEQGGITSES-SQNLRPRRANREKIENLLKSYSKRKPR 2035

PbCRMP4 ------------------------------------------------------------

PfCRMP4 TKDMTELNDFTTYYNEEINTYNIIQSSFSYHSDIKDISTTNENIEKYEHMQLLLQNRGNT 2245

PvCRMP4 KTFFYNYFMEEALFSNRDTLPTGFSTDTMGYKTGDENGGDDED----KENDAEINRLMDE 2029

PkCRMP4 KTFFYNYFMEEALFSQRDALPTGFSSDIMGYKTDEENGPDDEDDENDKENDDEINRLMDE 2095

PbCRMP4 ------------------------------------------------------------

PfCRMP4 PNIFENYFYQKDVY---------------------------------KKNNMSIYNMNLY 2272

PvCRMP4 GMRRMIEQKEKEQEEKAENGGEEKDAKERDAKEKVDEEKDAKEEDAKEEDAKESDDEENE 2089

PkCRMP4 EMKKKKEEMDKEAKKKAK-----KKKEEMDKAEKGGEAKDDKHKGVRDMDDKQNNNYKHN 2150

PbCRMP4 ------------------------------------------------------------

PfCRMP4 NMSPYVVYNNNKKKITN------------------------------------------- 2289

PvCRMP4 EEEYADDENEEEEYADDEKDDESKEREDKGGKQRVFFDNDDKEDKIRGYSEGHYLSQLDN 2149

PkCRMP4 EE---------------------------G-------------DKFIGYGEDKYMQGEDD 2170

PbCRMP4 ------------------------------------------------------------

PfCRMP4 ------------------------------------------------------------

PvCRMP4 KGKNKNKNRTGVKHLLIYITRAIRKKIIIALSKFYEGVQMEPQEIMYYKRKSGATVNYIL 2209

PkCRMP4 Q--NDDDGSQGAKHIFLYITKAIRKKMKISLSKFYEGIKLDDKEIMYYKRKGGATVNYIL 2228

PbCRMP4 ---------------------------------FYDIQSIDVNELVYYKNSQSIFANFNL 1889

PfCRMP4 --------------------IQVKNRLKIILTKMYHMTSVDINEVIYYKGKGELLTDLNL 2329

:*. .:: :*::*** . .: *

PvCRMP4 DSITLK-NDLSIFVCCYEGDFLESLPIEQNSYPNINKTYKVYLLYKVLSLYFYNINENIE 2268

PkCRMP4 DSIVLR-NNLSIVACCYEGDYVESIPITEGNY-YVRKYPTVYLLYKALKIYFGKVDENIT 2286

PbCRMP4 ENIIII-NAKKVIFCCQEEEYFEVEIVDDNKY-VIKKVSKIQQLLNKFKNYNAQLEKNIK 1947

PfCRMP4 QNIFILPKQKKVILCCYNDDYIEVIINDDNKI-GMKKSNKIQELIDILKKYHPNFERNKK 2388

:.* : : .:. ** : ::.* :.. :.* .: * . :. * :.:.*

PvCRMP4 GNNSALEQLNNAHDVYHEYKYLRIENLK----EKGFYKNQTLVQLKDTTFELKIQGKNIP 2324

PkCRMP4 GNNTLLQHLPNTYDGYHQYNYLRIENLN----ERGFYKNQTLAQLKDTEFELKIHGQNIP 2342

PbCRMP4 DQNLILEQFTNLYYKNNRPHYLMIKDST----DIGFYKNHMVIKLAEYNFSIQIKGENIP 2003

PfCRMP4 KQNDILEQFNNINDILFKYDYIRDVNKNQIYYDTGFFRNNIYIQLINQPFDLKIYGSNIQ 2448

:* *::: * . .*: : . : **::*: :* : *.::* *.**

PvCRMP4 ---PHEYTLYIVEFDNNCYR-LDKNGYHVENNEYT-LHSNYIIFKNVLMNKRNTYKLCIL 2379

PkCRMP4 ---PHEYTLYIVEFDNNCYR-LNKNGYHVENNKYT-IQSNYIIFKNVIVRKRNTYKLCIL 2397

PbCRMP4 ---DHKFILYIIEFNNSCYT-IDKKLFYVEKSEFSNKNSKSITFKDIQISKINNYKLCIL 2059

PfCRMP4 KKKTKDYILYIIEFHNICYNNMNKNGFFLEHKRYN-EMENYIHFLQISMNKINNYKLCLY 2507

:.: ***:**.* ** ::*: :.:*:..:. .: * * :: : * *.****:

PvCRMP4 DKTQDTYNDVGILNINNYYVKLES-FFD-------------------------------- 2406

PkCRMP4 DKTQDTYNDVGILNINNYYVKLES-FFD-------------------------------- 2424

PbCRMP4 DKTENIYTDVGILNITNYYIVNDS-YFD-------------------------------- 2086

PfCRMP4 DKERDIYSNVGILNITDYFVIKESSFFDNEDIGNIKKKNMETDSYNYNNDNNYDGNYDNN 2567

** .: *.:******.:*:: :* :**

PvCRMP4 -ENFISSNLKKMN-----------IMLNVC--VNNNSYDAYVIKRKGSFKITLDMEKLAF 2452

PkCRMP4 -ENFISSNLKKMN-----------IILNVC--VNNKSYDAYVIKRKGSFKIPLDMQKLAF 2470

PbCRMP4 -DDNIPSTSNQNNY----------AILNVC--VNIQYNNVYIIKKSRSFYFEIDSKNLTF 2133

PfCRMP4 YDNNYDNNYDQNNNKYYIKNYNQDVIKNVCLIMDNNTNQIIVIKGKGSFYFNLQIEKGIF 2627

:: .. .: * : *** :: : : :** . ** : :: :: *

PvCRMP4 S-DKSQQLEEIFPEIRNHQLISCKSKKHGTIILTNTHLFFYAL-----VTKLSFSTNHNL 2506

PkCRMP4 S-GKGQPLDEFFTGIQNHQLISCKSKEHGTIILTKTHLFFYAL-----VTKLSFSTDHNL 2524

PbCRMP4 L-KKNNNFNKYFPELQQHQLISCKSKDKYTIILTKTNVFLYILNHNNNTSELSFSTSHNI 2192

PfCRMP4 IEKKGQKLNELFPDIKNHEPISCKNKKKNTIILTKTHIFFYTN-----NNDISFSTKHNL 2682

*.: ::: *. :::*: ****.*.: *****:*::*:* ..:****.**:

PvCRMP4 PFPVDVDFDESYIYVTDLNLRKIARFQIFSEG---SAINIINNA-KRKRSIHDQDLRAYK 2562

PkCRMP4 PFPVDVDFDERYIYVTDLNLRRIARFQIFSQG---NAINIINKG-KRKRSIQDKDLRTYK 2580

PbCRMP4 MFAVDIDFDDNYIYISDLHLKKIIRLQIMING---IPTSIVNK--RSKRHIIGVDQSNIK 2247

PfCRMP4 PFPYDIDFDDQYIYVNDLYLNKIARIQILSNKDNKTPIPIINNKQRSKRSIINHGHESQQ 2742

*. *:***: ***:.** *.:* *:**: : . *:*: : ** * . . :

PvCRMP4 R-NTPMGEQGTTSKNITLFYPKEGRNFYTT-DLVPSP--LHGAVYPYGARNFKTALLTSK 2618

PkCRMP4 R-NTPIIEQGRTDKKINVYYPKEGRNFYNT-DLVRDA--IHGDIYSDDAKIFKTTLLTNK 2636

PbCRMP4 LLQNKILNKGKLLKSYVMHAKTSDENHLQTNHLVNSSN-IYDAQHYNRFDVLFPKLLINK 2306

PfCRMP4 N----IQKTHQTYDHFTPLHKVEKKEILQNNNIVQLKENEFPITNLKNIEYSKNNIPNQK 2798

: : . . .: . .:* . : .*

PvCRMP4 EAPKKEAIVLTKRSKQS---ADSYTENAH--QNLLQLRMLRRDMRNEKE----KTARLRR 2669

PkCRMP4 EAPKSDATVLTKRGIRS---GDSYAEKDH--QNLLHFPTLGRDMRSEKE----KISRLRR 2687

PbCRMP4 NSHKFEQLKLKK------------SKKSE--TNNFNKQTMRTNLNYNPQ----NTQNPKQ 2348

PfCRMP4 ISSKDDIYKMSDFQAFINKYANLITTNSFRLYNYFEKKKYYGTLNYFIKRVWFNKVGPSR 2858

: * : :.. : : * :. :. : : :

PvCRMP4 AEQSAPHNHPRGQQIKLDANPNVDASKKKPKSVANKEENALVKNVR-ELYNYLQKKVDNN 2728

PkCRMP4 AEESVFHSEQRTKQIKLDDNPIVDASKKKNLLVENKDENTFVKNVK-DLYSYLLSMGSKN 2746

PbCRMP4 NNEIVTFLQQYN-SYAVSNNVKKNFLKDENIQMNYR-ESSMSKIIS-KLYNYLKKTVSNI 2405

PfCRMP4 EENYSTESKIEQNDKNNNNNNNNNINNNYNYNYNDLHNNNHNIILNSNNYNNNNQSSRHQ 2918

:: . . . * : :. :. : . *. . :

PvCRMP4 GLINYFHGLLGPQKISKRRNSMYSFLDFFKNGKKKNLSSGNKNEVHSQIVPSS-MPARAK 2787

PkCRMP4 GLTNYFYGLLGPEKFSKKKNFMSSFLDFFKSEKKTNRSSGNENEIHRQIVPSS-MSARAK 2805

PbCRMP4 SSSVYLYKILDNKNILKEKSIISNLLNLFKKSKKKMNS--YDNNAMSLKIPDS-IIVHEE 2462

PfCRMP4 NFIYSYHVSLDGKKKQSENLLTHSLNNFIQFFQNKKNSILKKEKEKRKNIPSYNIMYKNI 2978

. : *. :: ... .: :::: ::. * .:: :*. : :

PvCRMP4 KQARRPVPRSENAARPRITVARK-KRDATEN-VDHSVYDEEIEKNIKNLITVVQTSNYLT 2845

PkCRMP4 KNVIRRVRRS--------TVGRK-KRDATKS-VNHSVYDEAIERNIKNLITVVQSNNYLT 2855

PbCRMP4 KNVSNNPNIMNTTS----ELTRK-KRNALQK-LDQSDYDKIIQKNIENFVNLPNTEKYLP 2516

PfCRMP4 NHITNTLNKRKQQELTSSIIKKRTKRNVQEKRMSQSAYNELVLASIKKYTNKVEPAHFVN 3038

:: . : :: **:. :. :.:* *:: : .*:: . :. :::

PvCRMP4 NQMEHLD-LKGHQSPLFISIQDNILYILDSTKNNFFSYNLYSKRIIQLEQYSAANHKFFL 2904

PkCRMP4 NKMDHLD-LKGYQNPLFISIQDDKLFILDSTENNFFSYNLKSKEIIQLEQYSATEHKFSL 2914

PbCRMP4 NNMSYLD-IANLKSPLHISIHDNILYILDRTKNNFFSYNLKSKKIIELEKYNFSTHKFTL 2575

PfCRMP4 NKLDYLNYITNLKSPLNISVQNNILYILDSIKNTFFSYNLINKEIKELEKYDFNDHPLIL 3098

*::.:*: : . :.** **:::: *:*** :*.****** .*.* :**:*. * : *

PvCRMP4 KNPLNFSIYRGEKTVHTPYRTNVAFVTQIRSNEIKIIDLNKNK-YRIVKAIRLEKGY-IH 2962

PkCRMP4 KNPLHFSIYQGEKTEHTLHRTNVAFVTQIRSNEIKIIDLSKSK-YRIVKAIRLEKGY-RH 2972

PbCRMP4 NNPLNFSIYEGNKIIHTEHTNKLAFVSQINSNEVKIIDLNQNQ-YKIIKAIKLRKGYKKH 2634

PfCRMP4 KNPLHFSIYNGIITEYTEHKNNLAFVTQIDSNEIKVIDLNSSKKYKIIKAIKLRKGY-KH 3157

:***:****.* :* : .::***:** ***:*:***...: *:*:***:*.*** *

PvCRMP4 NAVNKVIAIDQNLLIITSH-INAENII---YHYHFNSFDQYFSISFEYTFAKNYNSNDYL 3018

PkCRMP4 NGVSKVIAIDQNLLIITSH-INAENII---YHYHFNSFDQYFRISFEYTFAKNYNSNDYL 3028

PbCRMP4 RIINKVIPINETLLIITSQDLNDNGITPHHYHYYFNLFSHYFQISFSYTFSKRYTANDHL 2694

PfCRMP4 NHINYVIGIKQKLLIITSQSQNIDHYKNIIYHYHFNSFDQYFSISFNYTFAKNYNANDNL 3217

. :. ** *.:.******: * : ***:** *.:** ***.***:*.*.:** *

PvCRMP4 NIAPEIDIYSDSVIFFCFLNSFDLCTKVDPLTKLEISTETGVITGKLKHFGYFQLKIFAK 3078

PkCRMP4 NVAPEIDIYSDSLIFFCFLNSNDQCTNVDPLTKLEISSETGIITGKLKHFGYFQLKIFAK 3088

PbCRMP4 HIIPEIDKNSDTIIYFCFLNSSNKCTNVDELTKLKISYNTGIITGKLNYFGTFKLTIFAK 2754

PfCRMP4 YITPDIDKYSDNIIYFCFLNSNNKCTNIDELTKLSISHETGIITGKLNYFGTFSLKIFAK 3277

: *:** **.:*:****** : **::* ****.** :**:*****::** *.*.****

PvCRMP4 THFQHQVKVYTDLYSYCDVGKQYNYANQTCESCPIGTFWNSVELKCDLCKKYFKNRSTLK 3138

PkCRMP4 THFQHQVKLYKDLYSYCDVGKQFNYKNQTCESCPIGTFWDSVELKCESCKKHFKNTSTRK 3148

PbCRMP4 TYFQYKINIYDNLYSYCGIGKKFNINNNGCEGCPIGTFWNNEMLLCEKCDGYKKNTSTLK 2814

PfCRMP4 TYFQYKINTYSNLYSYCGISQEYNKTKSTCESCPIGTFWNSEKLLCENCQKYIENTSTLK 3337

*:**:::: * :*****.:.:::* :. **.*******:. * *: *. : :* ** *

PvCRMP4 RGSKLITDCLCSAGYEYSEKTASCEQCKPGYYKKSTGNFICIKGCPINRKSTIYGAKSYN 3198

PkCRMP4 SGSKLITDCLCSAGYEYSEKTASCEQCKPGYYKKSAGNFICIKGCPINRRSTIYGATSYN 3208

PbCRMP4 IGSKLITDCLCSAGYEYSEKTHSCEQCKPGYYKNRVGNFICIKGCKINETSIIYGATNYD 2874

PfCRMP4 NGSKLKTDCLCSPGYEYSDKSSKCEKCKPGYYKKSVGNFICIKGCPINEKSITYGAKSYG 3397

**** ******.*****:*: .**:*******: .********* **. * ***..*.

PvCRMP4 QMNCKCSEGYYELNGKCVLCLKNYYCPGNDQLIHCPKNKMSPEGITSADECVCKRNFIHN 3258

PkCRMP4 EMNCKCIQGYYEHNGKCVLCLKNYYCPGNNQLIPCHKNKMSPEGVTSADDCVCKENFIYN 3268

PbCRMP4 EMSCKCVEGYYMNNQKCTLCLKDHYCPGNEVIIQCNQNETSQIGSKLKENCVCKENFIYN 2934

PfCRMP4 EMDCKCIEGYYRKEDKCVLCLSNYYCPGNELIKPCDKNKISKEGVIHKNDCICKENYIKD 3457

:*.*** :*** : **.***.::*****: : * :*: * * ::*:**.*:* :

PvCRMP4 ENNQSCTYCTSVGKVKDEVIYCSLCDPRYLDT----NVFAIPND-HNYGLVDKIFNYDED 3313

PkCRMP4 ENNQSCTYCTSVGKVKDQVIYCSLCDPKYLDT----NIFAIPNN-HNYGLVNNIYNYDED 3323

PbCRMP4 KENNNCTYCVSIAKVKSEPIYCKLCEEKYLNI----NIFAVPYQ-NNYGLVNTIHNYYED 2989

PfCRMP4 NKNNNCVYCASIAKILGKPIYCSLCDPIYFHEDIYKNIFLLPNNIHNYGLINNIYNYDED 3517

::*:.*.**.*:.*: .: ***.**: *:. *:* :* : :****::.*.** **

PvCRMP4 LLLQGKTLQYAYFDNNG-RKNGRENGNKNNPLNDPPNDRKIDHAN-FGTLNINSELVLGN 3371

PkCRMP4 LLLQDKILQYSYFNKNG-PKVGRAIDRADDCANDRANGCAIDRTN-FGTLNINRELVLGN 3381

PbCRMP4 FILDKENVRYAYFGKKLEREENREIQIDSKIPRNYAN--YINFIK-FNDHDNNKNDALDG 3046

PfCRMP4 LSLQGNIMKYAYFHKTT------------NIERNFTH--YINIKNIYHTFNNNIN----- 3558

: *: : ::*:** :. . .: .: *: : : : * :

PvCRMP4 NPDEPNRETFAIEIDMPKPTKCVFCESGYFMDIKKERCLPCSNKYCEGFAKNPKGCPTNS 3431

PkCRMP4 NEDEPERETLAIEVDMPKPSKCVFCESGYFMDPKKERCIPCSNKYCEGFAKYQKGCPINS 3441

PbCRMP4 GNDAKDTIKFAIEVNTHHHTNCVFCESGHFIDYKKNKCLPCSNKYCKGFSKEPKNCPNNS 3106

PfCRMP4 NYDYLTNSKFAIEIDIPEPKKCIFCDSGYYI--KKKKCISCTTKYCEGFNHNPKACPKNS 3616

. * .:***:: . .:*:**:**::: **::*:.*:.***:** : * ** **

PvCRMP4 VVSRTVASSIFDCKCKIGYGSINERRSALDKKLSCKLCPKNFFSHNLSEQYCLPCPQHTY 3491

PkCRMP4 IVSGTMASSIFDCKCKIGYGSINERRSAFNKKLSCKLCPKNFFNHKLSEQYCLPCPQNTY 3501

PbCRMP4 IVNKKEASSIFDCVCKRGYGTLNERRNGIKKSLTCKVCPKNFFKHNTTDEYCLPCPYKTY 3166

PfCRMP4 VVNKMKASSIFDCSCKRGYGILNERRNAYSNTITCQICPQNFFKHIISDDSCFPCPLYTY 3676

:*. ******* ** *** :****.. .:.::*::**:***.* ::: *:*** **

PvCRMP4 TVSEGSTSILNCLPVEGYFLLMFRSIGIEYVRYKFDINSEDRIYSYFQDSVKSFDDDVYE 3551

PkCRMP4 TLAEGSTSILNCLPEDGYFLLMFKGITIEYMRYKFDINSENRIYSYFHDSVKSFDQDVYD 3561

PbCRMP4 TLLEGSNSISNCLPREGHFILMLRNINIEYLRYKFDSNMENTIYNYFQNNNKSFDDDVYD 3226

PfCRMP4 TLNQGATSILQCLPKKGYFIYMFKSINIDYLRYKFDINTENRIYNYFENNNKIFDEDVYN 3736

*: :*:.** :*** .*:*: *::.* *:*:***** * *: **.**.:. * **:***:

PvCRMP4 LFLDQKFEQLFNDADNNPDKRGQQNNEAVLPAKGQQNDGAVLPPNGQQNNGAALPANGQQ 3611

PkCRMP4 LFWEQNFQQIFNDVDNNPERKGHQNN--------Q------------------------- 3588

PbCRMP4 LFLNNNFEQVFQNLNNNTKTNNSINKK--------------------------------- 3253

PfCRMP4 FFLNKQYSKFFYNTENKTNTNNNHNNNNNNNHNHNNHNNN-------------------- 3776

:* ::::.:.* : :*:.. .. *:

PvCRMP4 NNGAALPANGQQNNGAALPPEGQYNAQNAQNAQNEQNAPNA-AN--APNAQDNKYNLEHL 3668

PkCRMP4 ---------------APLPPD-------------EKN-----------NAQNNMYKLKHL 3609

PbCRMP4 ----------NGNKEEKLYPK----------EDNDQNVINNNPN--NQNNNNNKYNLKDL 3291

PfCRMP4 ------HNNHNNNNNNKYTKDN--------HFDNQVGNINKHTNNLQIKITENIYNIQYL 3822

. : . : :* *::: *

PvCRMP4 KKMGFLKRTSLFYIYTVLMKNIEENYDWESDKMLKVTCHVNLHLKSSPNFTISYKPNLQS 3728

PkCRMP4 KKLGFLKRTSLFYIYTVLLKNIEENYVWESDKMLKVTCHVNLHLKSSPNFTVSYKPNLKS 3669

PbCRMP4 KKLGFLEPNVIYYIYSIMLKNIEDNYNWENDNNLNVICHINVHSKNNPNFTVTYTPNLNS 3351

PfCRMP4 EHLGFIKKTSLFYIYMILLRNIEERYNFNMDKMLKITCHINLHLKKNPHFTITYKKNLQE 3882

:::**:: . ::*** ::::***:.* :: *: *:: **:*:* *..*:**::*. **:.

PvCRMP4 CVDSCKTNIYCTGVEFSRKKVEYTQIFLKNNQKIIVGYFKCKHYSFESIYDYTSPNVDEF 3788

PkCRMP4 CVDSCKMNIYCTGVEFSRKKVEYTQIFLKNNQKIILGYFKCKQYSFESIYDYTSSDVDNF 3729

PbCRMP4 CISSCKSNIYCTGVEFSRYRVEYEQIFLKNNQKIIVGYFKCKKFYYKDIYYYSSPNLEQF 3411

PfCRMP4 CIDSCKTNIYCTGIEFSRKRVEYTQIILKNNKKIILGYFKCKQFYFQNIYDYSSYNLDSY 3942

*:.*** ******:**** :*** **:****:***:******:: ::.** *:* :::.:

PvCRMP4 FDENVENAVVSRTPVAYTDFILRNKNSIIFTCSIDRDRKFLLYKQYQPVGCFMGKFCPGN 3848

PkCRMP4 WDENIENAVVSKTPLAYTDFILRNKNNIIFTCSIDRDKKFLLYKKYQVAGCFMGKFCPGN 3789

PbCRMP4 LNENVQNGVPIKNPLAYKNFILGYKNKTIFTCSIDRDKKYLLYKKYQITECFAGKFCPGN 3471

PfCRMP4 LNEHIENQINIKSTLTYTNFIMRNKPDIIYTCSIDRDPKYLLYKNYKLVECVIGKFCPGN 4002

:*:::* : :..::*.:**: * . *:******* *:****:*: . *. *******

PvCRMP4 YTPYLISCPDNSKTVVTLAKHVDKCLCMKGYSYVGIATLRCAVCERGTYKKNVGNFKCEN 3908

PkCRMP4 YTPYLISCPDNSKTVVTLAKHVDKCLCIEGYSYVGIATLRCAVCDRGTFKNNVGNIKCEN 3849

PbCRMP4 NVPYLISCPNNSTTIVKLTSTVDKCLCMEGYAYLGLISHRCSICERGTYKNTIGNIKCEN 3531

PfCRMP4 NTPYMISCPINSTTIIPASEDVNKCLCLKGYSYVGVTSYRCSLCDRGSYKNSVGNNKCDN 4062

.**:**** **.*:: :. *:****::**:*:*: : **::*:**::*:.:** **:*

PvCRMP4 CPLTFSTVNIGSTSIYDCSCTPGNYLSFDTLQNFINSEKINYKNIIDQYFIVPKKKQT-- 3966

PkCRMP4 CPLTFSTVNIGSTSINHCSCTPGNYFTFDTLQNFINSEKINYKNIIDQYFIVPKKKQT-- 3907

PbCRMP4 CPLTFSTIKIGSKNISDCSCTLGNYIEFDTLGNFIKAENIIYKN-IDQYFIVPQKTDD-- 3588

PfCRMP4 CPLTFSTTHRGSTNIKDCSCTSGNYLTFDTLGNFIRTENMQHKNISDQYFVIPIKQESQP 4122

******* : **..* .**** ***: **** ***.:*:: :** ****::* * :

PvCRMP4 -----------DVY-----------YRTEIVKISLNNLNRIEVNEGTNQTLKREIAVCRP 4004

PkCRMP4 -----------DVY-----------YTTEIVKISLNNLNRIEVNEGTNQTLKREIAVCKP 3945

PbCRMP4 -----------NIY-----------YMTKAIKISLHNLNKKDDTEYINQILKLHIAVCRP 3626

PfCRMP4 ENEIKDDNKNINIYNANNINNHDIVYKTKMIKINLHKEND-NHNDYMNNILKQTIGLCKP 4181

::* * *: :**.*:: * : .: *: ** *.:*:*

PvCRMP4 CSLDNHYCEGGLESDITLNNKRFQGQFHTLPKKCPNDLVIPKGIIQRSSINNCICIHGKF 4064

PkCRMP4 CSLDNHYCEGGIESDITFNNRHFQGQFHTLPKKCPNDLVIPKGIMQRSSIKNCICIHGKF 4005

PbCRMP4 CSLDNHYCEGGIESNVVFNNKLINNNIHTLPKKCPNELVIPQGIKQRSSIKNCLCVHGKV 3686

PfCRMP4 CTLENHYCEGGLESDITFNNKLIKGKFHTLPKKCPRGLGIPNIIKERNSINNCLCIHGKV 4241

*:*:*******:**::.:**: ::.::********. * **: * :*.**:**:*:***.

PvCRMP4 LRTSKDKKVECIPCPPNTFKENEYDNSCSGVCPHFSTTFIGSSYENQCFCKNSYYLVTTE 4124

PkCRMP4 LRTSKDKQVECIPCPPNTFKESEYDNSCSGVCPHFSTTFVGSSYENQCFCKNNYYLVTTE 4065

PbCRMP4 LRELKDNTFECFPCPPNTFKESEYDNSCSGVCPHFSTSFIGSSFENQCFCKNNYYLTTAK 3746

PfCRMP4 QRTTENKNVECFPCPPNTFKENEYDNSCSGACPPFSTTFVGSSYENQCFCKNNYYLVSTD 4301

* ::: .**:*********.********.** ***:*:***:********.***.::.

PvCRMP4 ---------------------------DDNDGENSANRMESSKRTKSCKPCPKGAVCNRG 4157

PkCRMP4 ---------------------------DKNGWVYTTSTPKSGKGTKSCKPCPTGALCNRG 4098

PbCRMP4 ---------------------------INN--------QKNSNIKKNCELCPTGAICNRG 3771

PfCRMP4 DENDTNGNSINNNKNIYNNNNIYNNNNNNNNNNNNNFNNKSDVKKKSCKICPIGAICNRG 4361

.* :.. .*.*: ** **:****

PvCRMP4 FNINLFLHLLKNRAYNNINILDHENPYPLYGHYAVYKEK-HPISDWNPLED-----NDE- 4210

PkCRMP4 FNINLFIHLLKNREYNNINILDHENPYPLYGNYAVYKEK-HPISDWDPLQD-----NDE- 4151

PbCRMP4 FNIDLFIKLLKDRTYNNISILDHENPYPVYGYYAVYKRN-NKNTLWTPMDDKNSSSDDES 3830

PfCRMP4 FNIYIYLKLLNDRSYNNINVIDHETPFPKYGYYAVYREKTNPTKSWDTLDD-----SAI- 4415

*** ::::**::* ****.::***.*:* ** ****:.: : . * .::* .

PvCRMP4 ------------LKYSYPYYNLLLFIQKNVESGNYFFFEKNPNLYLNMEFIESIKKKKAN 4258

PkCRMP4 ------------LKYSYPYYNLLLFISKNVESRNYFFFKKSPKLYLNMDFIEALKKKKEN 4199

PbCRMP4 TNNNTKNNTDANFKYTYPYYNLILYIQKHVQNMNYFFFENNNKFYLDKTFIESVKNKNEM 3890

PfCRMP4 ------------LNYVYPYHKLLLFIQKNIQKGNYFFLEKGNKFFLNDDFVEKMNEKSKY 4463

::* ***::*:*:*.*:::. ****:::. :::*: *:* :::*.

PvCRMP4 KKATNAAEADEADEAHNPLFHQANAISILK---------YALKDSKSVKNPNEVIPNKHT 4309

PkCRMP4 KNETSPVRT---GEAPNFLLHRKNGTSSLN---------YNQNNGKAVKISNDVISNNHM 4247

PbCRMP4 KKKNNKHVD--ATESINDIYNQYNTISFIQNENKSDTSKYQNDQSHNNRKEKNIYTKQNK 3948

PfCRMP4 EEENANKFV-----TKSEIIHNVNTNNLKGKHN-----TLNNNNNNNINKSSSFLNQNNY 4513

:: . : . : :. * . .:.: . ... :::

PvCRMP4 N----AIYTYLK---KMVPTTLLAKPEPRPTPPLLQS-NEMEYS-------FQEMFNKYI 4354

PkCRMP4 N----AISTYFK---EIVPTILLVKPEPKPISPLQQS-NEMKYS-------FEEMFNKYV 4292

PbCRMP4 N----SNTTHDP---TKVDFYNLFSMQNKYLSFLESNKNMIKDTNKDTNNLIKEMFDNNL 4001

PfCRMP4 NNDIIQLSSYENNPFKNLSSTSEVSSNNNVISFIQNNDKTNNSTNDNNIENLVDIFNKYV 4573

* :: : . : . . : .. : : : : ::*:: :

PvCRMP4 EEANELRKKNNKFERLPDIHVCTLPDRCLGTITNLCQEGSTGYQCNNCQKGYDMSHFKSN 4414

PkCRMP4 EEANELRIKNNKFERLPDIHACTIPDRCLGTISNMCQEGSTGYQCNNCQKGYDMSYFKSK 4352

PbCRMP4 KEAKKITERNKQFERTPDIHPCNLPDRCLGTITNLCYEGSTGYQCNSCSKNYDMKYFKSK 4061

PfCRMP4 QEAKELQIQNRKFERIPDIHPCTIPNRCLGTLINLCSDGSTGYQCNNCSKNYDMSYFKSK 4633

:**::: :*.:*** **** *.:*:*****: *:* :********.*.*.***.:***:

PvCRMP4 CNRCKNFFNEILLMVLLKMIYYIIIIVIVSLNYNSCFNKLYISGILMKIWFNGSFTLVAY 4474

PkCRMP4 CHRCKNFFNEILLIILLKMIYYVIIILIVSLNYNSCFNKLYISGILMKIWFNGSFTLVAY 4412

PbCRMP4 CTKCRNIYHEILSIILLKIIYYVIIIYIVSLNYNSCLNNLYVSGVLFRIWLNCSFSFISL 4121

PfCRMP4 CKKCQNLFYEILHFILLKLLYYIIVIFICSLNGN-ILNKLHVSGILMRIWLNCSFTFIAY 4692

* :*:*:: *** ::***::**:*:* * *** * :*:*::**:*::**:* **::::

PvCRMP4 GFFSPTANSFITKYWHIYKSVFLSHLKMFSYYLRVACFAHSYNTDMRYNEIWYIQKYVNI 4534

PkCRMP4 GFFSPTANLFITKYWHIYKSVFLFHLKMFSYYLRVGCFFNLYNTSVHYNEIWYIQKYVNI 4472

PbCRMP4 GFFSPNNLSFITRYWYVYKEIFLYHLNFCSPYIRVGCFMSYYNTDITYKNIWYIQKYLNI 4181

PfCRMP4 GFFIPNMNSFISKYWYLYKTAFSYHMKIFAYYLRVQCLLNSLNINLSYNQMWYIQKYMNI 4752

*** *. **::**::** * *::: : *:** *: * .: *:::******:**

PvCRMP4 FTPLFDAISITLIMFVILHVYKLFCKKK-IQNFEHILSTIPELKAKYHLDCL-------- 4585

PkCRMP4 FTPLFDALSITLIMFFILHLYKFICKKK-IQNFEYILSTIPQLKVKYHLDFL-------- 4523

PbCRMP4 FTFFFDVFFITLILFIIMKITNFWHRKK-IQNFEIMLSTVPEVYDKFKIE---------- 4230

PfCRMP4 CTPIFDSIFITILLFAVIHIYKFFFYKKNIDIFEQLLCTIPELNTLYKKNCIDNIHKVKN 4812

* :** : **:::* :::: :: ** *: ** :*.*:*:: :: :

PvCRMP4 ----------------TKDPGGKASPKKGKKSKIYVSNKTEEGDNQDSCNHST------- 4622

PkCRMP4 ----------------TNNPDGKEFPKKGKKSKIYVCNKKKE-DDQDSCNNSS------- 4559

PbCRMP4 ----------------QND---KKKIKNKKTPKIHILNSNHEDQEDGSQNNLT------- 4264

PfCRMP4 KKFANKVESFEENENQNKEKKNKKNIKRKNKAKNKHNNDNNDDNDNNNDNNNDNNKININ 4872

:: * *. :..* *...: :::.. *:

PvCRMP4 ----VTHDLEKN-GVSD-----------KENAGETNGK-------SALIDENAPSVP--- 4656

PkCRMP4 ----LSQDLEKN-GISD-----------KENVLKPNDQ-------FTPIGRNAPYVP--- 4593

PbCRMP4 ----LIHDLEKNESQSDEYATHGDAQNFQNNRNDCNSQDEQGEEPHTLKNNTNVSVEKKI 4320

PfCRMP4 SKDEHINHFEKCSSITTILN------NLELNNEEKNKESKK---KKIFDIKKKTSIE--- 4920

:.:** . : : * . * : .. :

PvCRMP4 NLTNKKDAQVFCE--DDVLHKVDGDDPN--GECPKYELKYLNRDDKMYDIFLHETVEYIY 4712

PkCRMP4 NLTNRKNVQVFCE--DNILQNAYGDNQN--DEETKYELKYLKNDDKMYDIFLNETVEYIY 4649

PbCRMP4 NFLTKNKKSMLNN--SQIENKLTEENEN--IHDENYELKYIKKEHTMYSQFLNEAVEYIY 4376

PfCRMP4 NIKENETNHLWNDTHNNVKNKESNINTTNISNNINYELKYIKNKE-WYNIFLNKTIQYIY 4979

*: .: : : .:: :: : . . :*****::... *. **:::::***

PvCRMP4 N-KKIFGPWRFMHKRNEKLGRRLLTFLHDTFPCYILLIALSSPYVLMEVIQLAFCKPIKY 4771

PkCRMP4 N-KKIFGPWRFMHKRNEKLTRRLLTFLHDTFPCYILLMVLSSPYILMEGIQLAFCKPIRY 4708

PbCRMP4 D-KKMFGPWRFIHKKNDCFRKRFLGFISDSIPCYILMIIISTPYILLETVQLFYCKSIKF 4435

PfCRMP4 KEKKIFGLWHFIFTKNDNWFKNMGTFLNDTLPCYVLIMILSIPYILLELVQLTYCTPIKY 5039

. **:** *:*:..:*: :.: *: *::***:*:: :* **:*:* :** :*..*::

PvCRMP4 KSQESELYLSYLTTQKCTLSDSSFFTGIMVVILALFFYVGIFTLLITLYSKRNKYKLFNI 4831

PkCRMP4 KTQESELYLSYLTTQKCTLSDNSFFTGILVFILALFVYVGIFTLLIIIYWKRNKYKFFDI 4768

PbCRMP4 KSEKSELYLAYLNTQKCTTSSASFVMGLIVAFVVLLFYIATLILLLYLYSKRKTIKLFNK 4495

PfCRMP4 KTQKTYFYLTYLITQKCSLSSKLFITGLIVTITMFALYIFLWILLLYLYSKRKRLNLFDL 5099

*:::: :**:** ****: *. *. *::* : : .*: **: :* **: ::*:

PvCRMP4 LLSNLLTGYKQGKEIFEFIFILKNIILVLLITFSIHHEFYYVILITVILTTFSILEIYSN 4891

PkCRMP4 LLSNLLAGYKGGMEIFEFIFILKNIILALIVTFSIHYDFYYVILITVILTPFSILEIYST 4828

PbCRMP4 FLKNLLSGYRQGKEIFEVIFLLKNIILVVMIAFTIYYQYYYIVLITLTLTLFSILELISD 4555

PfCRMP4 LTTNLLSGFRKGKGIFELIFLLKHILIVLLICFNIYYGLYYIVFLTLILITFAILEILSN 5159

: .***:*:: * ***.**:**:*::.::: *.*:: **::::*: * *:***: *

PvCRMP4 PFDGRSFNILNISLCLGNVLNIFVALTIWGSFYWNYEQFFFVPILIILFYHLYMLHKIIR 4951

PkCRMP4 PFDGRSFNILNISLRIGNVLNIFVALTIWGSFYWNYDNFFFIPIFIILFYHLYMLHKVIR 4888

PbCRMP4 PFDRRSFNILNISLRVGSVLNIFFSIMIWGSFYLNYERHILFPFFIVILYHFYMVHNIVK 4615

PfCRMP4 PYDKRSFNILNKSLNIGNVLNIFFPLIIWGSFYWTYENFFIFPIFVIYAYHIYMFYHIVK 5219

*:* ******* ** :*.*****..: ****** .*:..::.*:::: **:**.:::::

PvCRMP4 EIILSRHFIRTENIISIKTAGYGESNIKHNNYKLYYKVMNKIKFFLNKNQKKNILSKQSP 5011

PkCRMP4 EIMLSRHTIHTENFINIKTDMYGENNIKDNNYKLYYRLMNKVKFFLNKNQKRNI-SKENA 4947

PbCRMP4 EVILSKYFITIQTYINVQTMGYDDHTIKNKNYKFYYKVMNKIKFVLKKKHKESILQYAK- 4674

PfCRMP4 EFTLSIHFIKTENIVNIQTIGYDPENIKNKNYKIYYKVMNKIKYFLNKNQKKNISNINEN 5279

*. ** : * :. :.::* *. .**.:***:**::***:*:.*:*::*..* . .

PvCRMP4 NNDGPNKINRMSRENIKFEPIENIIKRYNINLNDDLSKAKNLN----PNEEEEK------ 5061

PkCRMP4 NYDGPSKTNRMSKENIKFEPIENIIKRYNINLKEDISKAHSVN----NNEQVKM------ 4997

PbCRMP4 NDSSIEKTHGQPKEYIHFEAIDNIIKKYNDNLKKTVIRNIDQNEEIIDNEQKNI------ 4728

PfCRMP4 KDKHLNKNNN-SKDHVKFEPIENIIKRYNLKLNKGILKDANNNNN--NNNIKNMSDQVED 5336

: . .* : .:: ::**.*:****:** :*:. : : . * *: :

PvCRMP4 KKKEMFCIKKNHLPVNIHNVALIWYDEQNEDLLFQMPFENKFCLDLNGDK---------- 5111

PkCRMP4 KKKETFCIKKNRLPVNIHNVALIWYDEQNEDLLFQMPFENKFCLDLNGDN---------- 5047

PbCRMP4 KGKAQSSLKKKKLSVNVWNVAYIWYDEQNEDLFFQMPFEDKLSSSFNKE----------- 4777

PfCRMP4 NKKDILFIKKNVLPVNVHNVASIWYDEQNEDLLFQMPYQNKFCLNMNNEANENNDQINDL 5396

: * :**: *.**: *** **********:****:::*:. .:* :

PvCRMP4 -----------------------------AKNKKKK------------------------ 5118

PkCRMP4 -----------------------------AKNNKNK------------------------ 5054

PbCRMP4 -----------------------------PNEKTYN------------------------ 4784

PfCRMP4 TKDKTSEEENNNDHVNDPTKDQTNDEKKKNKNKKNKNNNNNNNENNCHSNDNSNNNNNKE 5456

:::. :

PvCRMP4 -----------------------------FFSFFPNYARDKRRRKNIKYFVSAIIEIIDK 5149

PkCRMP4 -----------------------------FISFFPKYARDKRRKKNIKYFVSAIMEIIDK 5085

PbCRMP4 -----------------------------IFARIPDRIKDKETKYNIKYFVLAIIQVIDK 4815

PfCRMP4 VHKNDDNINHNNINNHNKSSNISTTNKKNYFNKFSDYIIDKRNKKSIKYFITSLMEVIEK 5516

: :.. **. : .****: :::::*:*

PvCRMP4 FLCASKMGSIYENWFDFSMRFAFVYISWIKKVNKTN-VVLPHNMEQLKMNIDQYVFFPLF 5208

PkCRMP4 FLCASKIGSIYENWFDFSMRFAFVYISWIKKINKTN-VVLPHNMDQLKMNIDQYVFFPLF 5144

PbCRMP4 FVVISKSCSIYENWFDFAMRFAFVYISWIKRINKKD-IIIPNTMDQLHAITDEYIYIPLF 4874

PfCRMP4 FLDSSTHGFIYENWFDFTIRFAFVYISWIKKCNKKNNIMLPMNIQQLHIHMDLFIFTPIF 5576

*: *. ********::***********: **.: :::* .::**: * ::: *:*

PvCRMP4 YK--CELINQERRKLSVSAN-----TPEDQPENSAPIEALEKKQSFENTEIKISRSYGSY 5261

PkCRMP4 YK--CELINQERRKVTVSPN-----IHGEQSVNSAPIEALEKKHSFENTEIKISRSYGSY 5197

PbCRMP4 YK--YEYFTRPKKAFYESPKEETNYAPNNYEEGKYRTETLEKMNSFQNTEIKISRSYGSY 4932

PfCRMP4 PKNNSTCINEQNIQNYSKKKKIYN-KINMYDEKIETGELLEKHNSFENTEIKISRSFGSH 5635

* :.. . . : * *** :**:*********:**:

PvCRMP4 NQMGSLKNRKHAQEKNNHIMNDNYSDDAPSDKDAPSKG--KNETRKSSEEQAQMGVLSYL 5319

PkCRMP4 NHMGSLKNRKHGQDRNNQAMNENYSDDAPSDKDTSSKG--KNETRKSSEEQAKMGVLSYL 5255

PbCRMP4 SKMNSLKNRKKGKETNDYFNEQNSREDSSNGNDTNSSNFVRKNTKKSKSEHFGNNSQSVK 4992

PfCRMP4 NYMPNIKNRKNNYDKKEDITSINDINVDIIKQDDQKEDSNINHLINEHNQKEPSQHIYNI 5695

. * .:****: : :: . * : :* ... :. :. .::

PvCRMP4 KKKEKKKKKTQAK-----LKKKSIKMDNPEIHN---HINHKDHS-YKFENIFNHSMDIFS 5370

PkCRMP4 KKKERKKKKAQEK-----LKKKSIKMDNPDISK---HMNQKDHSSYKFENIFNHSIDIFS 5307

PbCRMP4 SSKDDDNNSNEENSVIKYLNEKESRKRNKKKKNSNDDSNTANIAKKASLNTFNYSLDVFS 5052

PfCRMP4 NDKNIMDYNIDEN-----ISKNNEKEKHIKIPPK--NISITQKEYYNNNDIFNYSLDIFS 5748

..*: . . : : :.::. : : . . . : : **:*:*:**

PvCRMP4 SEMFNESTFNMLISLFELYIAMKTLKSMDMQSFTKLYQLYNEKFIRFEKSLVFYINKLRD 5430

PkCRMP4 SEMFNESSFDMLISLFELYIAMKTLKSLDMQSFTKLYQLYNKKFIRFEKSLVFYINKLKD 5367

PbCRMP4 SEMFNEANYHILISLFELYIAMNTLKTMNYHTFNKLYKLYNKKYIIFEKNLTFYINNLRN 5112

PfCRMP4 SEMFNEKMFHSIISLFELYMAIKTLKLMNPHIFNKLYQMYNKKIITFDTSVMFYINKLRD 5808

****** :. :*******:*::*** :: : *.***::**:* * *:..: ****:*::

PvCRMP4 EIKLETEEIDNEIKTGDTAEKMRVKNYYFYKEELNKRKKLEQELMSKIECLKDCIDVNEK 5490

PkCRMP4 EIKLETEEIDNETKTGDTAEKMRVKNYYFYKEELNKRKKLERELMSKIECLQDCIDVNEK 5427

PbCRMP4 EIKHEANEIDNEIKQENLIEKIKIQNYYYYKEELLKRKNIEKELILKIKNLEDCVEANKR 5172

PfCRMP4 DIKSEIEQIDKEINTGDTTEKERIQNYYYYKEELFKRKKLEEELLSKIKSLEECIEVNKR 5868

:** * ::**:* : : ** :::***:***** ***::*.**: **: *::*::.*::

PvCRMP4 SQSLRNVL--KNSRKSKPSDGILDEDIHYIFSVLQNEGTYKLDHSKTKEPNE 5540

PkCRMP4 SQSLRNIL--KNSRNTIPSDGKVDEDIHYIFSVLQNEGTYKLDHSKTKKVND 5477

PbCRMP4 SRAIRNFL--RNPRNNNEPAESLADDINFIFSFLHNEGTYKLDHSKKKE--- 5219

PfCRMP4 SQSLRNMLLTKKKKNNNLNNENLEEHINYIFSHLHNEGTYKLNHSQNMDKKN 5920

*:::**.* :: ::. : :.*::*** *:*******:**:. .
